# Inhibition of METTL3 Alleviates NLRP3 Inflammasome Activation via Increasing Ubiquitination of NEK7

**DOI:** 10.1002/advs.202308786

**Published:** 2024-05-02

**Authors:** Xinyi Zhou, Xiaoyu Yang, Shenzhen Huang, Guifeng Lin, Kexin Lei, Qian Wang, Weimin Lin, Hanwen Li, Xingying Qi, Dutmanee Seriwatanachai, Shengyong Yang, Bin Shao, Quan Yuan

**Affiliations:** ^1^ State Key Laboratory of Oral Diseases & National Center for Stomatology & National Clinical Research Center for Oral Diseases West China Hospital of Stomatology Sichuan University Chengdu 610041 China; ^2^ Department of Prosthodontics Shanghai Ninth People's Hospital Shanghai Jiao Tong University School of Medicine College of Stomatology National Center for Stomatology National Clinical Research Center for Oral Diseases Shanghai Key Laboratory of Stomatology Shanghai Research Institute of Stomatology Shanghai Jiao Tong University Shanghai 200011 China; ^3^ Henan Eye Institute Henan Eye Hospital and Henan Key Laboratory of Ophthalmology and Visual Science Henan Provincial People's Hospital People's Hospital of Zhengzhou University People's Hospital of Henan University Zhengzhou 450003 China; ^4^ State Key Laboratory of Biotherapy and Cancer Center West China Hospital Sichuan University Chengdu Sichuan 610041 China; ^5^ Shanghai Engineering Research Center of Tooth Restoration and Regeneration & Tongji Research Institute of Stomatology & Department of oral implantology Stomatological Hospital and Dental School Tongji University Shanghai 200072 China; ^6^ Department of Oral Biology Faculty of Dentistry Mahidol University Bangkok 10400 Thailand

**Keywords:** m^6^A modification, METTL3, periodontitis, pyroptosis, small‐molecule inhibitor

## Abstract

N6‐methyladenosine (m^6^A) modification, installed by METTL3‐METTL14 complex, is abundant and critical in eukaryotic mRNA. However, its role in oral mucosal immunity remains ambiguous. Periodontitis is a special but prevalent infectious disease characterized as hyperinflammation of oral mucosa and bone resorption. Here, it is reported that genetic deletion of *Mettl3* alleviates periodontal destruction via suppressing NLRP3 inflammasome activation. Mechanistically, the stability of *TNFAIP3* (also known as A20) transcript is significantly attenuated upon m^6^A modification. When silencing METTL3, accumulated TNFAIP3 functioning as a ubiquitin‐editing enzyme facilitates the ubiquitination of NEK7 [NIMA (never in mitosis gene a)‐related kinase 7], and subsequently impairs NLRP3 inflammasome assembly. Furtherly, Coptisine chloride, a natural small‐molecule, is discovered as a novel METTL3 inhibitor and performs therapeutic effect on periodontitis. The study unveils a previously unknown pathogenic mechanism of METTL3‐mediated m^6^A modifications in periodontitis and indicates METTL3 as a potential therapeutic target.

## Graphical abstract

N6‐adenosine‐methyltransferase‐like 3 (METTL3) mediated m^6^A modification plays a pivotal role in periodontitis. Mechanistically, METTL3 installs m^6^A modification on the TNFAIP3 transcript and facilitates its degradation. TNFAIP3 is found to bind with NEK7 and catalyze its ubiquitination, thus suppressing NLRP3 inflammasome assembly and activation. Meanwhile, the natural compound Coptisine chloride is discovered as a novel inhibitor of METTL3.

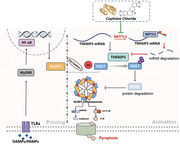

## 1 Introduction

Periodontitis is a common oral disease, which endangers both dental and systemic health.^[^ ^1^ ^]^ Periodontitis has become a major public health problem because poor periodontal conditions exacerbate the global burden of chronic diseases,^[^ ^2^, ^3^ ^]^ rheumatoid arthritis (RA),^[^ ^4^ ^]^ inflammatory bowel disease, and certain cancers.^[^ ^5^, ^6^, ^7^ ^]^ However, the intrinsic mechanism of periodontitis is not well defined, and there is limited access to targeted and effective therapeutic measures available to address its pathogenesis.

During the onset of bacterium‐induced periodontal diseases, human gingival tissues act as the frontline defense against pathogen invasion. Periodontal stromal cells, especially human gingival fibroblasts (HGFs), constitute the most abundant part of the human gingiva.^[^ ^8^, ^9^, ^10^ ^]^ Fibroblasts, in addition to serving as supportive cells for tissues, play immunological modulatory roles. In recent years, its diverse immunological properties have been elucidated, implicating its involvement in infectious diseases, chronic inflammation, and cancer progression.^[^ ^11^, ^12^ ^]^ To facilitate tissue maintenance and regeneration, fibroblasts react to pathogenic microbes and their products, recruiting immune cells and inducing the removal of damaged cells.^[^ ^13^, ^14^ ^]^ Evidence has increasingly elucidated the mutual interaction between GFs and immune cells in the context of periodontitis, which is facilitated through the utilization of intracellular inflammatory signaling pathways to initiate inflammation and drive connective tissue transmigration.^[^ ^8^, ^9^ ^]^

The discovery of epigenetic RNA modifications provides alternative insights into the processing of multiple cellular functions. As the most abundant post‐transcriptional modification of eukaryotic mRNAs, N6‐methyladenosine (m^6^A) is catalyzed by a multi‐subunit m^6^A “writer” complex containing the N6‐adenosine‐methyltransferase‐like 3 (METTL3)‐N6‐adenosine‐methyltransferase‐like 14 (METTL14) heterodimer and additional adaptors, among which METTL3 catalyzes m^6^A formation and installs modifications.^[^ ^15^, ^16^ ^]^ At the transcriptional level, METTL3‐mediated m^6^A modification can affect RNA stability, splicing, and translation. Recent studies have unveiled that METTL3 participates in numerous pivotal physiological and pathological processes, spanning from embryonic development to tumorigenesis and progression, which execute regulatory duties in cell cycle, apoptosis, differentiation, immune homeostasis and energy metabolism, etc.^[^ ^17^ ^]^

Meanwhile, METTL3‐mediated m^6^A methylation plays a direct and indispensable role in the inflammatory response. METTL3 enhances dendritic cells maturation by promoting the translation of target transcripts in the TLR4/NF‐κB pathway, including *Cd40*, *Cd80*, and *Tirap*.^[^ ^18^ ^]^ Moreover, METTL3 deficient macrophages exhibit the dampened capability to eliminate pathogens in an m^6^A‐dependent manner, mechanistically, the augmented expression of *Irakm* transcript without m^6^A modification impairs the TLR4 signaling pathway.^[^ ^19^ ^]^ Genetic silencing or pharmacological inhibition of METTL3 attenuates LPS and TNFα‐induced acute kidney injury via regulating the stability of *Tab3* transcript, an upstream regulatory molecule of NF‐κB.^[^ ^20^ ^]^ Taken together, these findings indicate that METTL3‐mediated m^6^A modification may be a key factor in the regulation of immune processes and inflammatory responses. Epigenetic networks govern inflammatory diseases like osteoarthritis, inflammatory bowel disease, acute kidney injury, pulpitis, and so forth,^[^ ^21^, ^22^, ^23^, ^24^, ^25^ ^]^ however, little is known about periodontitis. Our previous study suggested the involvement of m^6^A modification in periodontitis occurrence,^[^ ^26^ ^]^ but the exact mechanism remains elusive.

In this study, we discover that deletion of *Mettl3* alleviates periodontal destruction and inflammation. Mechanically, METTL3 deficiency attenuates NLRP3 inflammasome activation by impairing the degradation of *TNFAIP3* (also known as A20) transcripts. We further identify a selective natural small‐molecule inhibitor of METTL3, which displays promising therapeutic effects on periodontitis.

## 2 Results

### 2.1 Deletion of Mettl3 Ameliorates Periodontal Destruction

**Figure 1:**
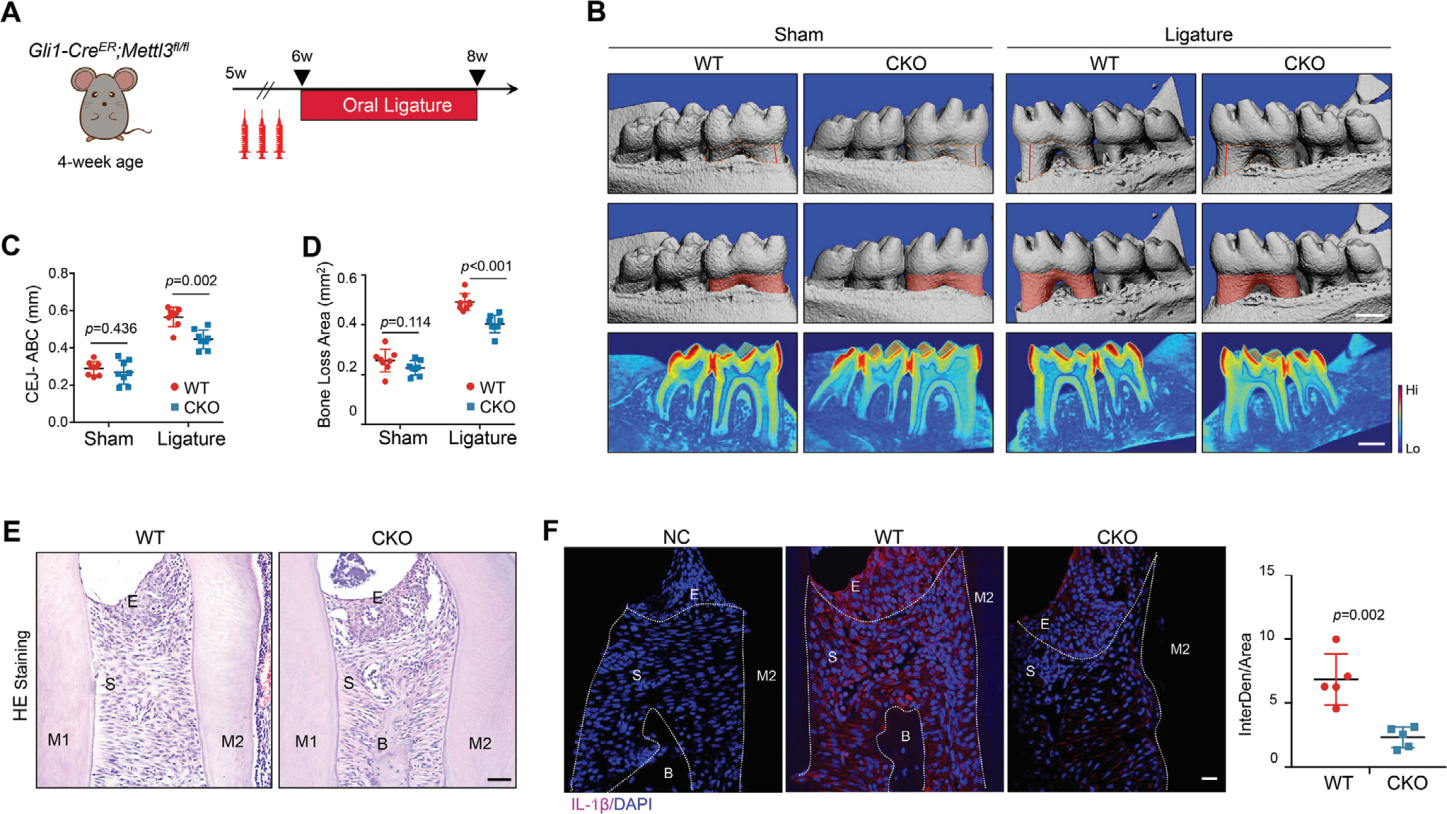
Deletion of *Mettl3* in periodontal mesenchymal cells ameliorates periodontal destruction. A) Schematic workflow of inducible knockout of *Mettl3* in periodontal stromal tissue and induction of periodontitis. B) Micro‐CT analysis and 3D reconstructed images observed from the lingual side. WT, wild type mice; CKO, *Gli1‐Cre^ER^; Mettl3^fl/fl^* mice. Images in the first line show the distance between the cemento‐enamel junction (CEJ) and the alveolar bone crest (ABC) as indicated by the red line on the M1 molar, signifying periodontal attachment loss. The upper dotted line indicates the CEJ and the lower one indicates the ABC. Images in the middle line show the area bordered by the CEJ, ABC, and the mesial and distal line angles of the M1 molar. Heatmap images in the last line evaluate the tissue density of 3D reconstruction. The right bar shows the density from high to low. Scale bars, 500 µm. C) Quantification of the distance between CEJ‐ABC. D) Bone loss area between M1 and M2 in WT and CKO mice (n = 8, one‐way ANOVA). E) Representative HE staining of WT and CKO mice with oral ligatures. E, epithelial layer; S, stromal layer; B, interdental alveolar bone. Scale bars, 20 µm. F) Representative IF staining of IL‐1β in periodontal tissues of WT and CKO mice with oral ligatures. NC, negative control. Scale bars, 20 µm. Results are shown as mean ± SD.

Gli1^+^ cells are multi‐potential stem cells that contribute to periodontal tissue homeostasis,^[^ ^27^ ^]^ and we generated *Gli1‐cre^ER^; Mettl3^fl/fl^* (CKO) mice to conditionally delete *Mettl3* in periodontal stromal tissues (Figure S1A, Supporting Information). The mice were subjected to intraperitoneal tamoxifen injections at 5 weeks of age for 3 consecutive days before oral ligation (**Figure** **1**A). We confirmed that Gli1^+^ cells contributed to most areas in the periodontal ligament and almost all gingival fibroblasts (Figure S1B, Supporting Information). Immunohistochemical staining verified a satisfactory knockout efficiency (Figure S1C, Supporting Information).

Periodontal insertion of ligatures induced robust alveolar bone destruction in wild‐type (WT) mice, while CKO mice exhibited less bone recession around the ligatures (Figure 1B–D). More remaining bone was observed in the furcation area of CKO mice compared with their littermates. Histological examination on the maximum coronal section of the first molar showed that alveolar bone crest resorption was much more severe in WT mice with ligation (Figure 1E). Immunofluorescence staining on the inflamed area between the first and second molar verified that deletion of *Mettl3* diminished the secretion of IL‐1β in gingival fibroblasts around the bone crest (Figure 1F).

As STM2457 is recently reported as a selective small‐molecular inhibitor of METTL3,^[^ ^28^ ^]^ we treated ligated mice with STM2457 and successfully ameliorated alveolar bone loss (Figure S2A–C, Supporting Information). Immunohistochemical staining of the periodontal tissues showed that GSDMD expression and IL‐1β production were decreased after STM2457 treatment (Figure S2D,E, Supporting Information).

### 2.2 METTL3 Regulates Inflammasome Assembly and Pyroptosis

**Figure 2:**
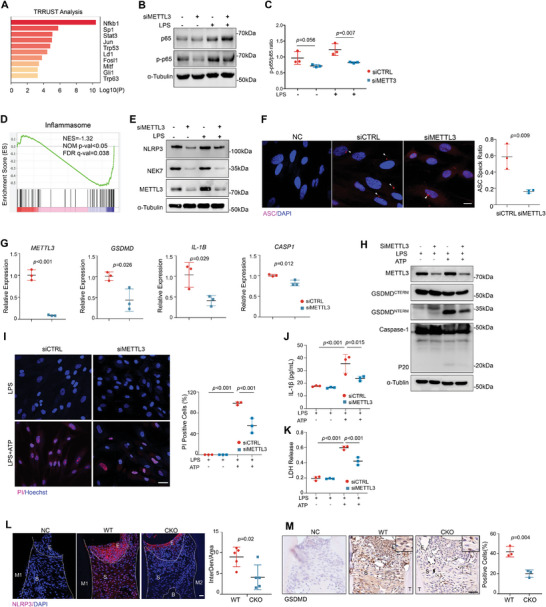
METTL3 regulates inflammasome assembly and pyroptosis. A) TRRUST analysis of genes downregulated in HGFs with METTL3 knockdown. HGFs were transfected with siCTRL or siMETTL3 and then treated with proinflammatory stimuli for 6 h. B,C) Western blot analysis of p65 and phosphorylated(p)‐p65 in HGFs with or without METTL3 knockdown in response to proinflammatory stimuli. D) Gene set enrichment analysis (GSEA) for genes associated with inflammasome in the siMETTL3 group versus the siCTRL group. E) Western blot analysis of NEK7, NLRP3, and METTL3 in HGFs transfected with or without siMETTL3. F) IF staining and quantification of ASC speck in proinflammatory stimuli treated HGFs (n = 3, unpaired two‐tail Student's *t*‐test). Scale bars, 10 µm. G) Quantitative real‐time PCR analysis of *METTL3*, *GSDMD*, *IL‐1B*, and *CASP1* (n = 3, unpaired two‐tail Student's *t*‐test). H) Western blot analysis of Caspase‐1 and GSDMD protein expression in HGFs. I) PI uptake staining and quantification of proinflammatory stimuli activated HGFs (n = 3, unpaired two‐tail Student's *t*‐test). Scale bars, 20 µm. J,K) Release of IL‐1β and LDH in cultured supernatants (n = 3, unpaired two‐tail Student's *t*‐test). L) Representative IF staining and quantification of NLRP3 expression in periodontal tissues of WT and CKO mice with oral ligatures. Purple signals are for NLRP3 positive staining and blue signals indicate DAPI. Scale bars, 20 µm. M) Representative IHC staining and quantification of GSDMD expression in periodontal tissues. Scale bars, 20 µm. Values are shown as mean ± SD.

We next performed RNA sequencing analysis to elucidate the underlying mechanisms and found that a total of 3338 transcripts were upregulated and 3131 were downregulated in small interfering RNAs (siRNA)‐mediated METTL3 deficient HGFs (Figure S3A, Supporting Information). In line with GO enrichment analysis, METTL3 deficiency led to a significant downregulation in inflammatory diseases including periodontal disease (Figure S3B,C, Supporting Information). TRRUST analysis of downregulated genes revealed that those transcripts were mainly enriched in the cluster regulated by *Nfkb1* (**Figure** **2**A). Next, we confirmed a down‐regulated pattern of the NF‐κB pathway by analyzing the phosphorylated(p)‐p65/p65 ratio in METTL3 deficient HGFs (Figure 2B,C).

As NF‐κB activation triggers inflammasome priming and assembly, we implemented GSEA analysis and verified that genes associated with the activation of inflammasome were significantly downregulated in METTL3 deficient HGFs in response to proinflammatory stimuli (Figure 2D). We further treated HGFs with LPS followed by ATP activation to induce NOD‐like inflammasome activation. Immunoblot assay revealed the expression level of NLRP3 and NEK7, was significantly reduced by silencing METTL3 (Figure 2E). Notably, the depletion of METTL3 hindered the ASC speck formation in HGFs (Figure 2F), indicating impaired inflammasome assembly and activation.

The expression of pyroptosis‐related transcripts including *GSDMD* and *CASP1* was also decreased (Figure 2G). As Caspase‐1 mediated pyroptosis is accomplished by the cleavage of pro‐Caspase‐1, we next evaluated the protein expression levels of p20 by immunoblot assay. The levels of the activated form of Caspase‐1, as well as the N‐terminal of GSDMD protein, were reduced after METTL3 knockdown (Figure 2H). PI uptake assay revealed fewer dead cells in METTL3 deficient HGFs (Figure 2I), implying METTL3 knockdown protects cells from pyroptotic death. As a result, cytokines maturation and membrane disruption were hampered in METTL3‐deficient cells thus IL‐1β secretion and LDH release were downregulated (Figure 2J,K). Histological analyses of mouse periodontitis models confirmed the expression of NLRP3 as well as GSDMD was downregulated in CKO mice (Figure 2L,M).

### 2.3 Disulfiram Administration Relieves Periodontitis in Mouse Models

**Figure 3:**
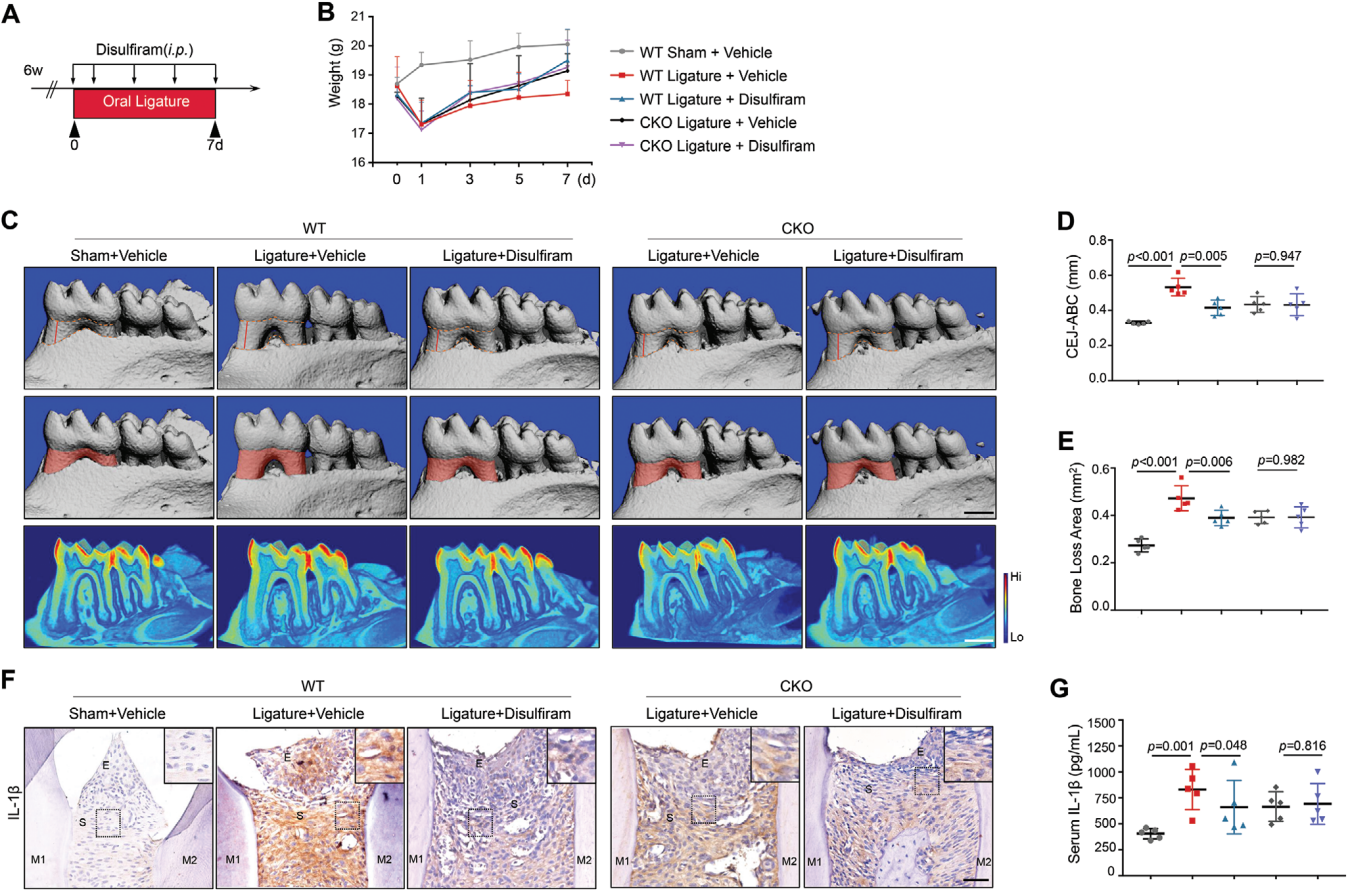
Administration of disulfiram relieves periodontal inflammation. A) Schematic workflow of the oral ligature and disulfiram administration. B) Body weight at indicated times. C) Micro‐CT analysis and 3D reconstructed images observed from the lingual side. Scale bar, 500 µm. D) Quantification of the distance between CEJ‐ABC (n = 5, one‐way ANOVA). E) Quantification of bone loss area (n = 5, one‐way ANOVA). F) Representative IHC staining and quantification of IL‐1β in periodontal tissues. E, epithelial layer; S, stromal layer. Scale bars, 20 µm. G) ELISA measurement of serum IL‐1β (n = 5, one‐way ANOVA).

To evaluate whether blockade of pyroptosis could ameliorate periodontal destruction, we treated ligated mice with disulfiram, a newly discovered pyroptosis inhibitor (**Figure** **3**A).^[^ ^29^ ^]^ The body weights of mice usually got rapidly dropped after oral ligation as periodontal trauma directly impaired dietary capability (Figure 3B). Disulfiram‐treated WT mice had increased body weights on the 7th day after ligation, suggesting improved recovery from masticatory disorders, while such effect was abolished in CKO mice. Notably, micro‐CT analysis revealed that disulfiram administration significantly alleviated periodontal bone absorption in WT mice (Figure 3C–E). Moreover, disulfiram treatment reduced the expression and maturation of Caspase‐1, IL‐1β, and GSDMD in the gingival tissues of WT mice (Figure 3F,G; Figure S4, Supporting Information). Interestingly, no significant difference in bone loss or tissue pyroptotic level was observed between CKO mice treated with or without disulfiram (Figure 3C–G), indicating the therapeutic effect of disulfiram on periodontitis was METTL3‐dependent.

### 2.4 METTL3‐Mediated m^6^A Promotes *TNFAIP3* Degradation

**Figure 4:**
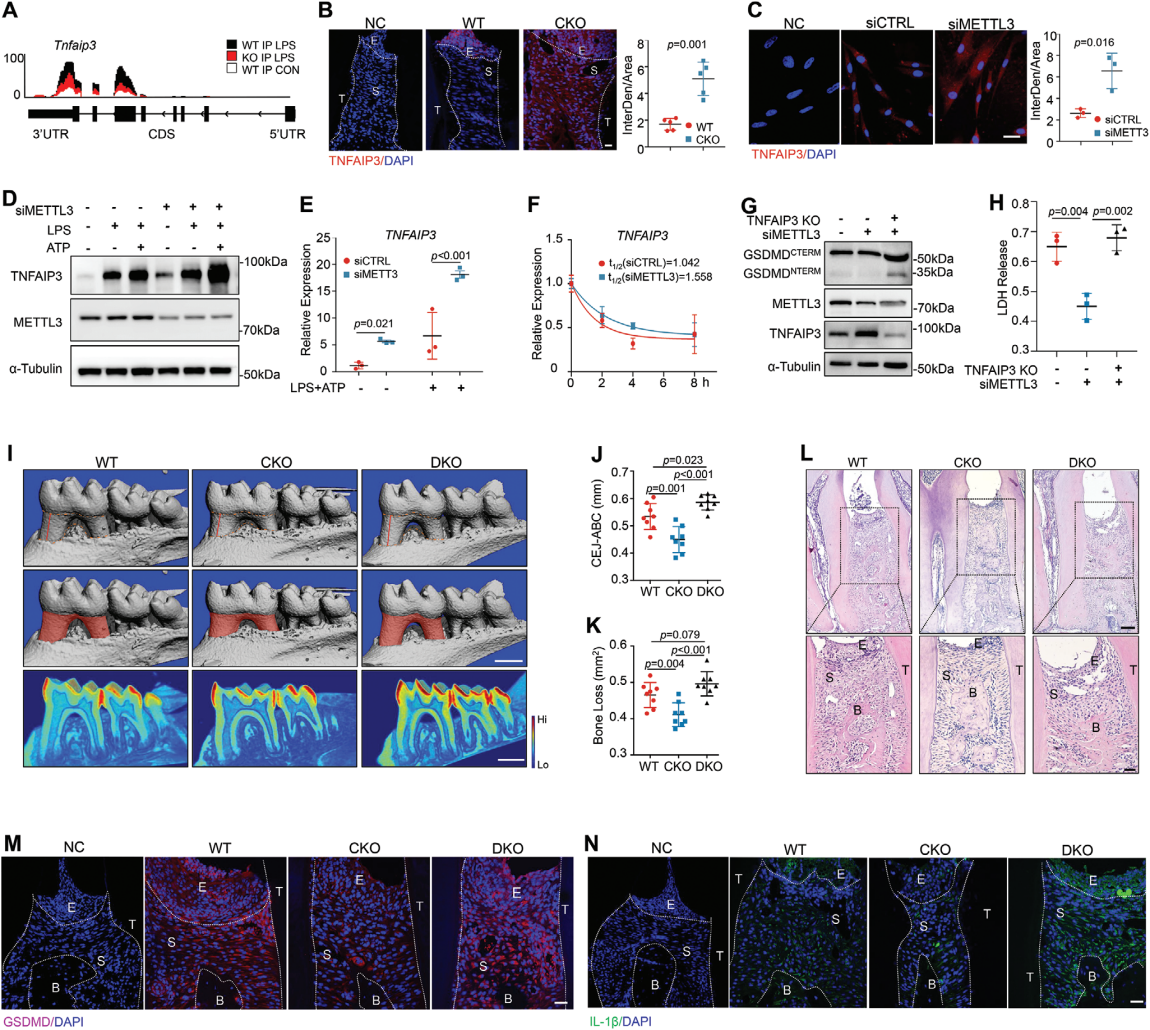
METTL3‐mediated m6A promotes TNFAIP3 degradation. A) MeRIP‐seq indicating m^6^A peaks enriched in the 3′UTR of *Tnfaip3* mRNAs. UTR, untranslational region; CDS, coding sequences; LPS, LPS‐treated; CON, control‐treated. B) Representative IF staining and quantification of TNFAIP3 expression in WT and CKO mice with ligatures (n = 5, unpaired two‐tail Student's t‐test). E, epithelial layer; S, stromal layer; T, tooth. Scale bar, 20 µm. C) Representative IF staining and quantification of TNFAIP3 expression in proinflammatory treated HGFs (n = 3, unpaired two‐tail Student's *t*‐test). Scale bar, 20 µm. D) Western blot analysis of TNFAIP3 and METTL3 in HGFs. E) Real‐time PCR analysis of relative TNFAIP3 expression in HGFs (n = 3, unpaired two‐tail Student's *t*‐test). F) The level of *TNFAIP3* in HGFs treated with actinomycin D (n = 3, unpaired two‐tail Student's *t*‐test). G) Western blot analysis of GSDMD, METTL3, and TNFAIP3 expression in HGFs after TNFAIP3 knockout and proinflammatory stimulation. H) LDH release assessed in HGFs supernatants (n = 3, unpaired two‐tail Student's *t*‐test). I) Micro‐CT analysis and 3D reconstructed images observed from lingual side. WT, wild type mice; CKO, *Gli1‐Cre^ER^; Mettl3^fl/fl^* mice; DKO, *Gli1‐Cre^ER^; Mettl3^fl/fl^;Tnfaip3^fl/fl^*. Scale bars, 500 µm. J) Quantification of the distance between CEJ‐ABC (n = 8, one‐way ANOVA). K) Quantification of bone loss area in 3 groups (n = 8, one‐way ANOVA). L) Representative HE staining of WT, CKO, and DKO mice with oral ligatures. E, epithelial layer; S, stromal layer; B, interdental alveolar bone; T, tooth. Scale bars, 20 µm. M) Representative IF staining and quantification of GSDMD expression in periodontal tissues of WT, CKO, and DKO mice with oral ligatures. Scale bars, 20 µm. N) Representative IF staining and quantification of IL‐1β expression in periodontal tissues from 3 groups. Scale bars, 20 µm. Results are shown as mean ± SD.

The function of METTL3‐mediated m^6^A modification relies heavily on the regions where the motifs located within transcripts.^[^ ^30^, ^31^ ^]^ Through analysis of published MeRIP sequencing data,^[^ ^19^, ^32^, ^33^ ^]^ we found TNFAIP3, a negative regulator of TNF‐α, had m^6^A modification peaks near the stop codon of its transcripts (**Figure** **4**A; Figure S5A, Supporting Information). Specific m^6^A peaks were observed on the 3′UTR region of *Tnfaip3* mRNAs upon LPS stimulation, and these effects were largely retarded by the deletion of *Mettl3* (Figure 4A).

Notably, deletion of *Mettl3* led to a higher level of TNFAIP3 in the gingiva of mice with oral ligatures (Figure 4B). Similarly, elevated TNFAIP3 protein level was detected in cultured METTL3‐deficient HGFs (Figure 4C–E). Moreover, proinflammatory stimulation robustly induced TNFAIP3 expression, and this effect was exacerbated upon METTL3 depletion (Figure 4D,E). Next, we performed RNA decay assays by treating HGFs with actinomycin D and assessed the abundance of *TNFAIP3* over time. The degrading rate of *TNFAIP3* in METTL3‐deficient HGFs was ≈1.5 times slower than the control (Figure 4F).

Further, we deleted *TNFAIP3* in the METTL3‐deficient HGFs and observed a higher expression of the activated N‐terminal of GSDMD (Figure 4G). LDH release was also significantly increased (Figure 4H), suggesting that loss of TNFAIP3 aggravated NLRP3 inflammasome activation. We subsequently generated *Gli1‐cre^ER^; Mettl3^fl/fl^;Tnfaip3^fl/fl^* double knockout (DKO) mice with simultaneous deletion of *Mettl3* and *Tnfaip3* in periodontal tissues. Micro‐CT analysis and HE staining showed aggravated destruction of the alveolar bone crest around the interdental area in the DKO group (Figure 4I–L). The number of GSDMD‐positive cells was largely increased in DKO mice when compared to that of CKO, and these cells mostly clustered around the alveolar bone crest where the gingival collagen bundle attached to stabilize the teeth (Figure 4M). IL‐1β positive granules were also elevated after *Tnfaip3* knockout, especially scattering in the cytoplasm of fibroblasts (Figure 4N).

### 2.5 TNFAIP3 Inhibits Pyroptosis by the Ubiquitination of NEK7

**Figure 5:**
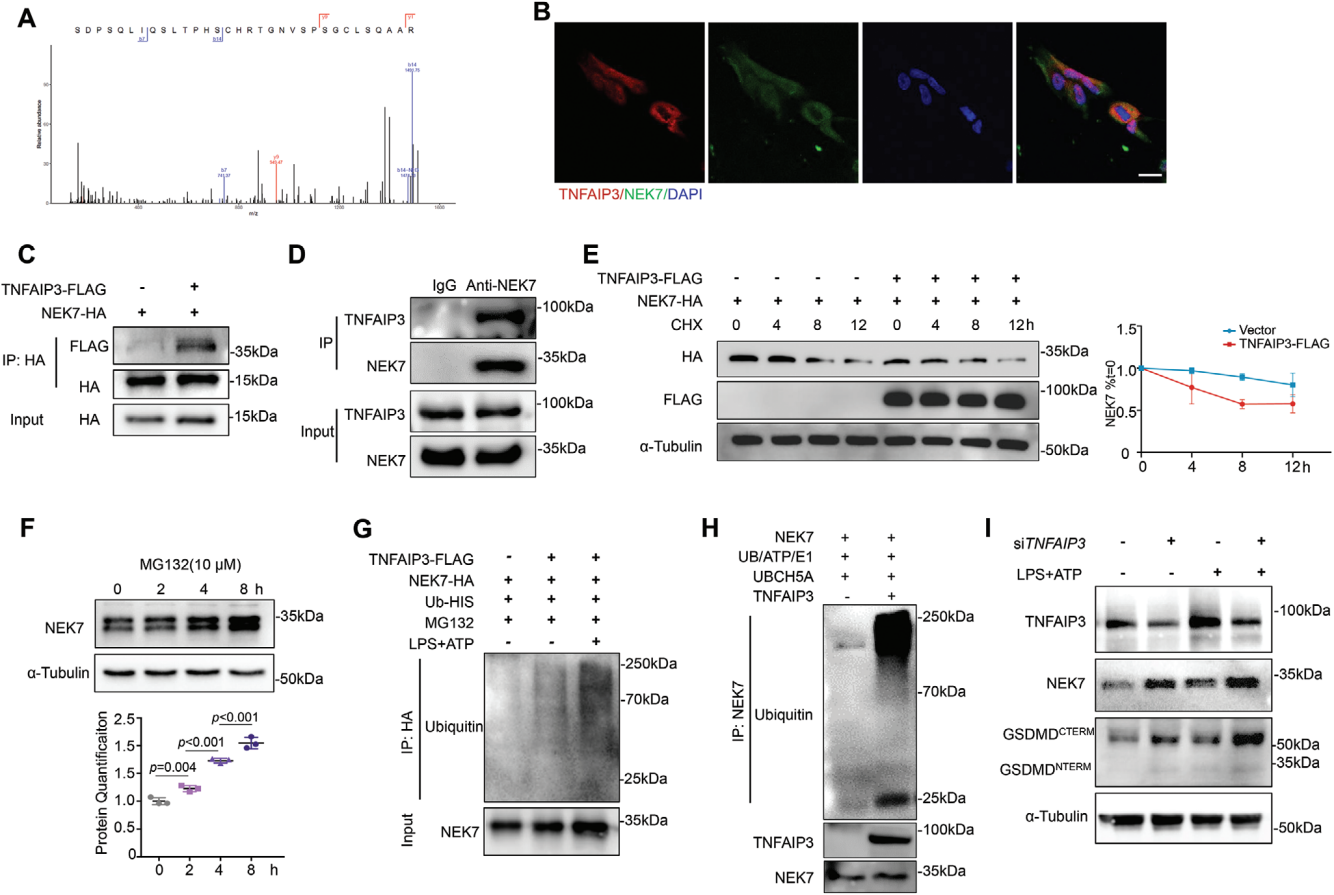
TNFAIP3 interacts with NEK7 and promotes its ubiquitination. A) Mass spectrometry analysis identifies the protein affinity of TNFAIP3 with NEK7 upon LPS and ATP stimulation. B) Representative IF staining showing the co‐location of TNFAIP3 and NEK7 in HGFs with proinflammatory treatment. Scale bars, 20 µm. C) Co‐IP analysis of TNFAIP3 in ectopically NEK7‐overexpressed HEK293T cells. D) Co‐IP analysis of TNFAIP3 and NEK7 in HGFs. E) Western blot and the quantification analysis of the degradation of NEK7 in the presence of TNFAIP3. F) Western blot analysis showing the expression level of NEK7 after proteasome inhibition (n = 3). G) Co‐IP analysis of NEK7‐linked ubiquitin. H) In‐vitro ubiquitylation analysis of TNFAIP3‐catalyzed ubiquitination of NEK7. I) Western blot analysis of TNFAIP3, NEK7, and GSDMD in HGFs transfected with siTNFAIP3 in response to proinflammatory stimuli. Values are shown as mean ± SD.

TNFAIP3 exerts marked effects in numerous inflammatory biological processes,^[^ ^34^, ^35^ ^]^ and previous data prompted the binding of NEK7 to TNFAIP3.^[^ ^36^ ^]^ To further validate such interaction, we conducted a mass spectrum assay (MS) of NEK7 immunocomplex to elucidate the potential binding molecules. TNFAIP3 was found to be among the top proteins that directly bound to NEK7 (**Figure** **5**A). Immunofluorescence staining confirmed the colocalization of NEK7 and TNFAIP3 in HGFs (Figure 5B). By transfecting TNFAIP3‐FLAG‐tagged and NEK7‐HA‐tagged plasmids into HEK293T cells, we verified the direct interaction between TNFAIP3 and NEK7 with a co‐immunoprecipitation (co‐IP) assay (Figure 5C). Endogenous co‐IP confirmed their direct binding within HGFs (Figure 5D).

To determine whether TNFAIP3 directly regulates the stability of NEK7 protein, we performed protein degradation assay by measuring NEK7‐HA expression levels in the presence of cycloheximide (CHX), an inhibitor of protein translation. Overexpression of TNFAIP3 resulted in a faster degradation rate of NEK7‐HA protein (Figure 5E). We also observed the proteasome‐dependent protein degradation of NEK7 was inhibited by MG132 treatment (Figure 5F). Notably, overexpression of TNFAIP3 increased the ubiquitination of NEK7, and it was further augmented after LPS and ATP treatment (Figure 5G). Additionally, we performed an in vitro ubiquitylation assay and substantiated that TNFAIP3 assumed the role of E3 to directly catalyze the ubiquitination of NEK7 (Figure 5H). In addition, silencing TNFAIP3 led to upregulated expression of NEK7 and GSDMD (Figure 5I), indicating an augmented response to inflammation on account of increased NEK7 expression.

Since canonical pyroptosis induced by NLRP3 inflammasome assembly and caspase‐1 activation was first found and mostly studied in macrophages, we verified the METTL3‐TNFAIP3‐NEK7 axis in immortalized bone marrow‐derived macrophage (iBMDMs). Similar to that in HGFs, the knockdown of METTL3 attenuated pyroptosis of iBMDM (Figure S6A–C, Supporting Information). Immunofluorescence staining of ASC specks showed that the assembly of NLRP3 inflammasome was suppressed (Figure S6D, Supporting Information). The transcription of *TNFAIP3* was increased in response to METTL3 knockdown (Figure S6A,E, Supporting Information), and the expression of NEK7 was suppressed. To evaluate the role of TNFAIP3 in NLRP3 inflammasome activation, we generated a TNFAIP3 KO cell line. Western blot analysis revealed that the expression of NEK7 and cleavage of GSDMD was enhanced in TNFAIP3 KO cells (Figure S6F, Supporting Information).

### 2.6 Discovery of Coptisine Chloride as a METTL3 Inhibitor

**Figure 6:**
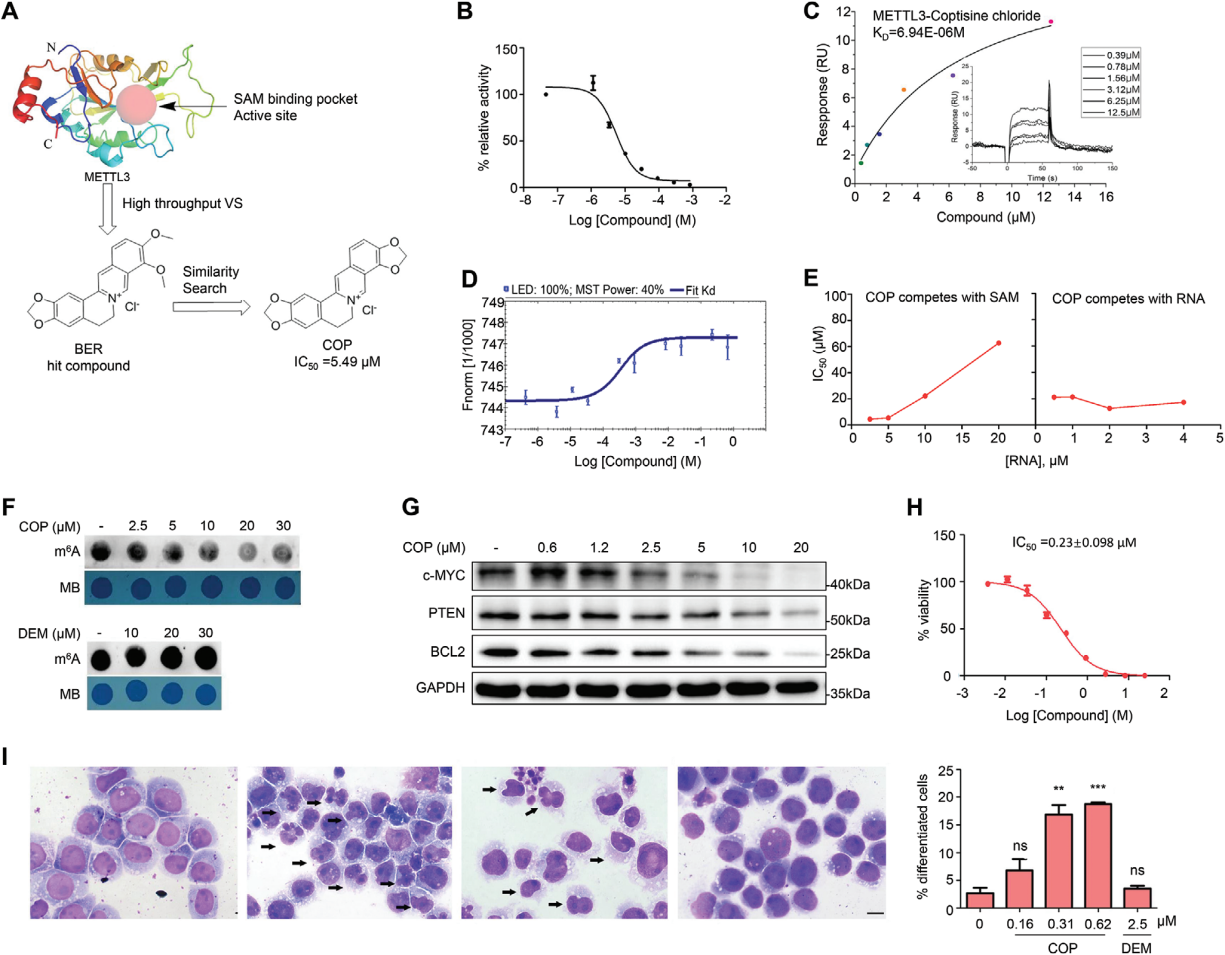
Discovery of a specific inhibitor of METTL3. A) Schematic diagram illustrating the discovery process of METTL3 inhibitors. B) Dose‐response profile of COP in the FRET‐based MazF assays. C) Representative METTL3 binding curves and fit steady‐state evaluation for COP using Surface Plasmon Resonance (SPR). D) The binding affinity of COP with METTL3 measured by Microscale Thermophoresis (MST). The error bars represent the mean ± SEM of each data point calculated from two independent thermophoresis measurements. E) Results of kinetic experiment. F) Dot blot of m^6^A levels of total RNA from MOLM‐13 cells treated with COP. G) Western blot analysis of c‐MYC, BCL‐2, and PTEN of MOLM‐13 treated with various COP concentration and time. H) MTT assay of MOLM‐13 viability after being treated with COP for 72 h. (n = 3). I) Representative images and quantitative analysis of Giemsa staining. MOLM‐13 cells were treated with DMSO (0.1%), COP or DEM for 5 days. (n = 3, one‐way ANOVA). Scale bars, 20 µm. ^*^ *P* < 0.05, ^**^ *P* < 0.01, ^***^ *P* < 0.001. ns, no significant difference between the control group and the treatment group.

To explore whether METTL3 is a potential therapeutic target for periodontitis, we sought to discover a small molecule inhibitor targeting METTL3 methyltransferase activity. We first performed a molecular docking‐based virtual screening against the commercial chemical library Vitas‐M (≈1.5 m compounds, Vitas‐M Laboratory, Ltd), and an in‐house chemical library (35000 compounds). We finally selected 30 agents from top‐ranked compounds (Table S1, Supporting Information). The differential scanning fluorimetry (DSF) assay showed that berberine hydrochloride (BER, **Figure** **6**A; Table S1, Supporting Information), a natural product, possessed the maximum △*T_m_* (△*T_m_* = 0.61 °C).

Next, we searched for analogues of BER from a commercial natural product library (≈1500 compounds) provided by Target Molecule Corp (http://www.targetmol.com). Six analogues of BER were retrieved (Figure S7A, Supporting Information): Coptisine chloride (COP), jatrorrhizine hydrochloride (JAT), palmaline chloride (PAL), dehydrocorydaline chloride (DEH), epiberberine chloride (EPI), and demethyleneberberine chloride (DEM). Both DSF and FRET‐based MazF assays showed that the most active compound corresponds to COP, while DEM did not display activity (Figure S7B,C, Supporting Information). The dose‐activity profile of COP was further measured, which gave an IC_50_ value of 5.49 µm (Figure 6B). In addition, Surface Plasmon Resonance (SPR) showed that the measured equilibrium dissociation constant (Kd) of COP was 6.94 µm (Figure 6C), while that of SAM was 15.2 µm (Figure S7D, Supporting Information). Very similar results were obtained in the Microscale Thermophoresis (MST) assays, which gave a Kd value of 0.244 µm for COP (Figure 6D). We then performed kinetic experiments to determine the mechanism of action (MOA) of COP. The IC_50_ values of COP increased with the increase of SAM concentration but did not change with the variation of RNA oligonucleotide concentrations (Figure 6E; Figure S7E,F, Supporting Information), indicating that COP is a SAM competitive inhibitor. Molecule docking predicted that COP occupies the SAM binding pocket (Figure S8A, Supporting Information). Three hydrogen bonds are formed between COP and METTL3 (Figure S8B, Supporting Information).

METTL3 is abundant in acute myeloid leukemia (AML) cells, and depletion of it prompts cell differentiation and apoptosis.^[^ ^37^ ^]^ To examine the inhibitory activity of COP in intact cells, we treated AML cell line MOLM‐13 with COP for 12 h. COP treatment resulted in a dose‐dependent decrease in the m^6^A level of mRNAs, while DEM, the negative control, did not show activity (Figure 6F). As the knockdown of METTL3 decreases the expression of BCL‐2, c‐MYC, and PTEN,^[^ ^38^ ^]^ we inspected the effect of COP on their expression. Western blot analysis demonstrated that COP effectively down‐regulated the protein levels of BCL‐2, c‐MYC, and PTEN dose‐dependently (Figure 6G). The protein levels of METTL3 and METTL14 were not affected by the COP treatment (Figure S7G, Supporting Information).

Finally, we performed MTT assay to test the anti‐viability activity of COP on AML cells, in which MOLM‐13 cells were treated by COP for 3 days. The results showed that COP significantly inhibited the survival of MOLM‐13 cells in a dose‐dependent manner (Figure 6H). Giemsa staining indicated that COP potently and dose‐dependently induced cell differentiation (Figure 6I).

### 2.7 Administration of Coptisine Chloride Ameliorates Periodontitis

**Figure 7:**
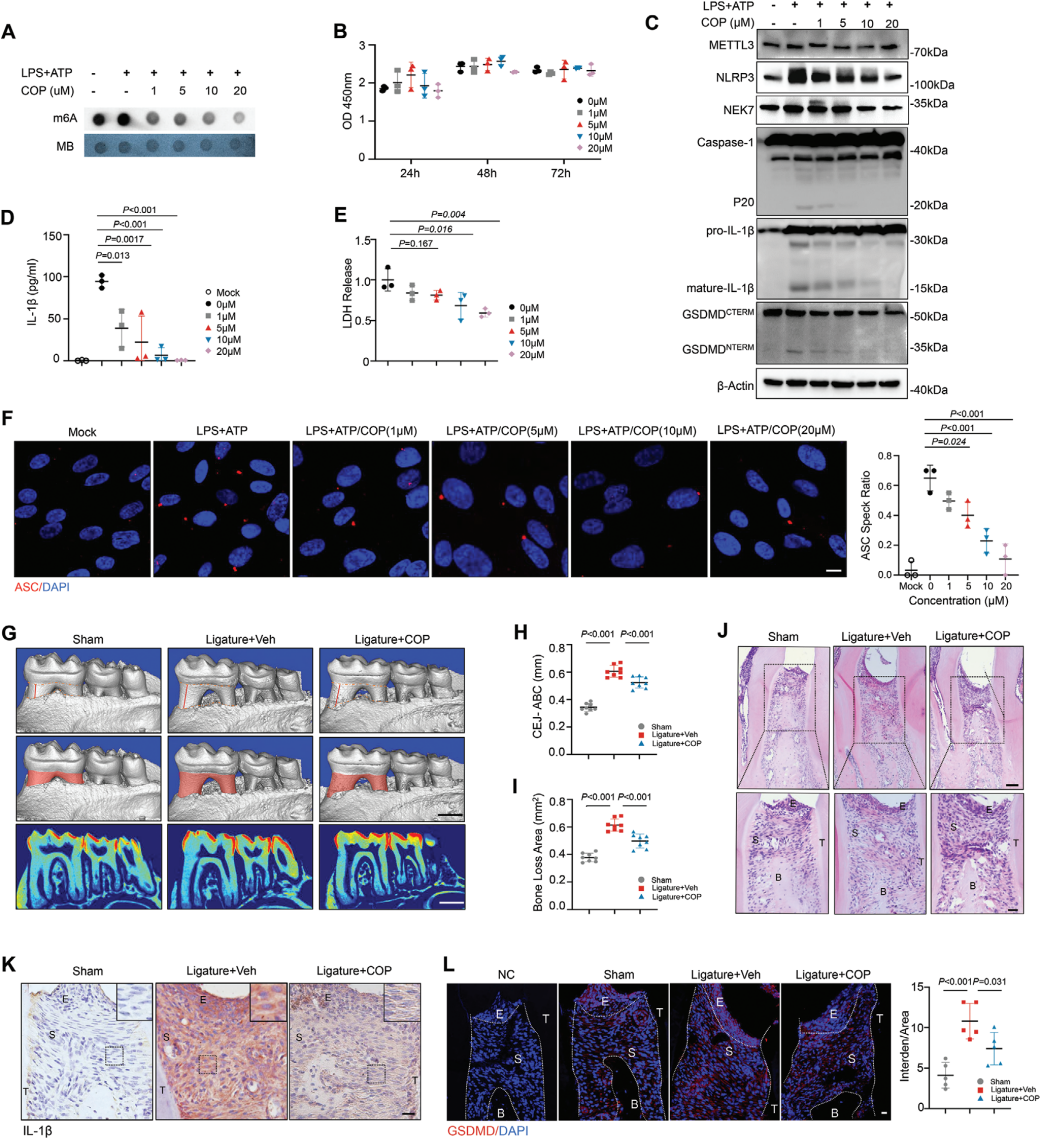
Therapeutic effect of Coptisine chloride on periodontitis. A) Dot blot analysis of m6A modification levels of HGFs with 0–20 µm COP treatment. B) CCK8 measurements of HGFs growth with COP treatment at 24, 48, 72 h (n = 3, one‐way ANOVA). C) Western blot of METTL3, NEK7, caspase‐1, IL‐1β, and GSDMD expression after COP treatment and proinflammatory stimulation. D) ELISA analysis of IL‐1β secretion (n = 3, one‐way ANOVA). E) LDH release of HGFs with 0–20 µm COP treatment under proinflammatory stimulation (n = 3, one‐way ANOVA). F) IF staining of ASC speck in HGFs treated with 0–20 µm COP (n = 3, one‐way ANOVA). Scale bars, 10 µm. G) Micro‐CT analysis and representative 3D reconstructed images observed from the lingual side. Scale bar, 500 µm. H) Quantification of the distance between CEJ‐ABC in 3 groups (n = 8, one‐way ANOVA). I) Quantification of bone loss area in 3 groups (n = 8, one‐way ANOVA). J) Representative HE staining of mice with oral ligatures received COP intervention or not. E, epithelial layer; S, stromal layer; B, interdental alveolar bone. Scale bars, 20 µm. K) Representative IHC staining of IL‐1β expression in local gingival. Scale bars, 20 µm. L) IF staining and quantification of GSDMD expression in local gingival. E, epithelial layer; S, stromal layer; B, interdental alveolar bone; T, tooth. Scale bars, 20 µm.

Next, we sought to evaluate the therapeutic efficacy of COP in preventing periodontal inflammation. We first confirmed that COP successfully decreased the RNA m^6^A levels of HGFs without obvious cytotoxicity (**Figure** **7**A,B). Notably, COP dose‐dependently downregulated the expression of NEK7, consequently suppressed maturation of Caspase‐1, as well as the cleavage of GSDMD and IL‐1β, while did not affected the expression of METTL3 (Figure 7C). Meanwhile, COP successfully reduced the release of IL‐1β and LDH (Figure 7D,E). Immunofluorescence staining revealed a decreased number of ASC specks in HGFs received COP treatment (Figure 7F).

Further, we performed daily injections of 50ul COP (20 µm) into the local buccal gingiva of periodontitis mice, starting from the day of periodontal ligation. Micro‐CT analysis presented that COP administration significantly ameliorated inflammatory periodontal bone loss (Figure 7G–I). Less tissue destruction and vertical alveolar bone resorption were observed in COP‐treated group (Figure 7J). Meanwhile, COP administration attenuated the expression of IL‐1β and GSDMD in periodontal tissues, indicating a relief of pyroptosis (Figure 7K,L).

## 3 Discussion

Pyroptotic cell death plays a crucial role in maintaining homeostasis in the immune environment, whereas critical signaling pathways that modulate inflammasome activation in the context of oral mucosal immunity lack further investigation. Here we identified METTL3 as a regulator of NLRP3 inflammasome activation in periodontitis. We demonstrated METTL3‐mediated m^6^A modification targeted *TNFAIP3* transcript and accelerated its degradation, resulting in the upregulation of NLRP3 inflammasome activation and pyroptosis. Interestingly, accumulating TNFAIP3 could boost the ubiquitination of NEK7, resulting in the hindrance of NLRP3 inflammasome assembly. These findings provide a previously undefined mechanism for post‐transcriptional regulation in oral mucosal immunity via the promotion of inflammasome formation. Furthermore, we explored a selective small‐molecule inhibitor of METTL3 and verified its inhibitory capacity on NLRP3‐mediated pyroptosis and periodontitis.

As a core methylation process, METTL3‐dependent m^6^A modification accounts for the most prevalent mRNA modifications in eukaryotic cells and has critical effects on many cellular processes, primarily by modulating the translation and stability of the modified mRNAs.^[^ ^39^ ^]^ Several pioneering studies have shown that a number of METTL3‐installed m^6^A‐modified transcripts are essential for innate and adaptive immune response.^[^ ^18^, ^19^, ^40^, ^41^, ^42^ ^]^ With respect to mucosal immunity, emerging evidences reported that under infectious conditions, intestinal epithelial cells exhibit increased global m^6^A abundance and display significant alterations in the topology of the methylome.^[^ ^43^, ^44^ ^]^ Accumulating evidence sheds light on the regulatory mechanisms of the epigenetic network in oral immunity. m^6^A modification affected dental pulp inflammation via regulating the splicing of MyD88 transcript, and knockdown of m^6^A demethylase FTO promoted inflammatory responses of cementoblasts.^[^ ^25^, ^45^ ^]^ Our previous study predicted the potential role of m^6^A‐associated single‐nucleotide polymorphisms (SNPs) in the pathogenesis of periodontitis.^[^ ^26^ ^]^ However, there is still a paucity of research regarding the precise role of the m^6^A modification in inflammatory oral diseases. In this study, we generate a murine periodontitis model by conditionally deleting *Mettl3* in periodontal mesenchymal cells. *Mettl3* depletion significantly decreased periodontal lesions and ameliorated local gingival inflammation, indicating the regulatory role of m^6^A modification in periodontitis.

Pyroptosis is a kind of programmed cell death involved in numerous inflammatory diseases,^[^ ^46^, ^47^ ^]^ which is initiated by inflammasomes assembly and characterized with membrane pore formation and inflammatory cytokines leakage. Pyroptosis was first reported in monocytes but was recently confirmed to occur in various cell types including fibroblasts.^[^ ^48^, ^49^ ^]^ Among the canonical inflammasome sensors that drive Caspase‐1‐mediated pyroptosis, the NLRP3 inflammasome has been extensively elucidated. Its dysregulation closely correlates with a variety of chronic low‐grade inflammations including periodontal diseases.^[^ ^50^, ^51^, ^52^, ^53^ ^]^ From a clinical aspect, NLRP3 inflammasome‐related proteins are also shown to have a positive association with the severity of periodontal lesions, and relevant markers are suggested as the indicators for periodontal prognosis.^[^ ^50^ ^]^ Genetic deletion of *Nlrp3* or drug inhibition of NLRP3 inflammasome formation could alleviate the progression of periodontitis in mice.^[^ ^54^ ^]^

Our data presented that silencing METTL3 suppressed the assembly of NLRP3 inflammasomes, and consequently restrained the expression and maturation of Caspase‐1, IL‐1β, and inflammatory cell death of HGFs. We administrated disulfiram to mice with periodontitis, a proved small‐molecular inhibitor of pyroptosis via inhibiting GSDMD cleavage.^[^ ^29^ ^]^ There was a rapid decrease in IL‐1β release in serum and in gingival tissue from periodontal‐ligated mice after disulfiram treatment, indicating the overall blockade of pyroptosis. Unlike the results in wild‐type mouse that disulfiram intervention slowed down the alveolar bone destruction progress in periodontitis mice, there was no obvious difference in CKO mice with or without disulfiram treatment, indicating that the regulatory effect of METTL3 in periodontitis worked through pyroptosis. Whereas disulfiram was reported will not affect either GSDMD or IL‐1β processing in vitro,^[^ ^29^ ^]^ our immunoblotting of extracted gingival tissues showed relatively downregulated expression of pro‐IL‐1β and GSDMD. We assumed this result could be attributed to the general suppression of IL‐1β release, and low circulating IL‐1β levels ameliorated the pro‐inflammatory cascades in gingival tissues.

The MeRIP‐seq data showed that *Tnfaip3* transcript is modified with m^6^A via METTL3. Interestingly, LPS stimulation exacerbated the abundance of m^6^A peak on the *Tnfaip3* transcripts in RAW 264.7 cells. TNFAIP3 functions as a critical gatekeeper in maintaining innate immunity homeostasis by establishing a negative feedback loop to regulate inflammatory cascades. It has been validated that TNFAIP3 can restrict pyroptosis induced by NLRP3 inflammasome activation independent of its inhibitory effect on the NF‐κB pathway.^[^ ^55^, ^56^ ^]^ In oral cavity, partial TNFAIP3 loss or impaired expression leads to increased gingival inflammation in oral mucosa.^[^ ^57^ ^]^ Intriguingly, TNFAIP3 is constitutively expressed in gingival connective tissues: its expression is relatively low under healthy conditions and modestly upregulated in the gingival tissue of periodontitis patients,^[^ ^58^ ^]^ indicating its sustainability may be altered by post‐transcriptional modification. In this study, we found that removal of METTL3/m^6^A modification resulted in increasing protein expression of TNFAIP3 via reduced mRNA degradation. In periodontitis models, knocking out *Tnfaip3* offset the protective effect of *Mettl3* depletion. Liu et al. reported that NEK7 can interact with TNFAIP3 but has no effect on TNFAIP3 phosphorylation in alveolar macrophages from pulmonary fibrosis patients. In our research, mass spectrometric analysis and co‐IP analysis confirmed the high affinity of NEK7 and TNFAIP3. Respecting the inner interaction of two molecules, overexpression TNFAIP3 significantly accelerated degradation of NEK7. Ubiquitylation is an universal mode of endogenous protein degradation. TNFAIP3 has been reported to possess ubiquitin‐editing enzymatic activity.^[^ ^59^ ^]^ We validated TNFAIP3 interacts with NEK7 and promotes its degradation in a ubiquitin‐dependent manner, afterwards restraining NEK7/NLRP3 activation.

As a bacterial infectious disease, clinical therapy of periodontitis for a long time mainly confined to the clearance of dental biofilm. However, active intervention toward tissue inflammatory reaction may be conducive to relieve periodontal bone resorption. Pharmaceutical development targeting epigenic modification has become a topic in regulating host cells biological process, for instance in antitumor immunity.^[^ ^60^ ^]^ STM2457 is a newly identified selective catalytic inhibitor of METTL3. Yonkava et al validated that treatment of AML with STM2457 reduced tumor growth and promoted cancer cells differentiation and apoptosis.^[^ ^28^ ^]^ Promising medical treatments that intervene rampant responses are in urgent need for periodontitis, so as to directly ameliorate tissue destruction. In this study, we performed high‐thorough screening and uncovered a novel small‐molecule inhibitor of METTL3 targeting SAM‐binding site, COP. Therapeutic studies demonstrated COP effectively inhibited NLRP3 inflammasome activation in vitro and attenuated periodontal inflammatory bone loss in vivo. COP is a natural compound isolated from traditional medicine Coptis chinensis, structurally similar to the well‐known Berberine. Although there is a higher inhibitory concentration of COP compared to STM2457, COP, as a natural product, exhibits promising biosafety.^[^ ^61^ ^]^ Meanwhile, previous studies have investigated its pharmacokinetics and tissue distribution.^[^ ^62^ ^]^ These properties may offer convenience for clinical application.

In summary, we find that deletion of *Mettl3* alleviates periodontal destruction via inhibiting NLRP3 inflammasome‐driven pyroptosis, and discover Coptisine chloride as a novel METTL3 inhibitor. Our study unveils a previously unknown pathogenic mechanism for periodontitis and indicates METTL3 as a potential therapeutic target, which may shed light on the clinical treatment of inflammatory diseases.

## 4 Experimental Section

### Antibodies and Reagents

Primary antibodies included anti‐IL‐1β (Abcam, ab254360), anti‐GSDMD (Abcam, ab219800), anti‐GSDMD (Huaan, HA721144), anti‐Caspase‐1 (Abcam, ab179515), anti‐Caspase‐1 (Adipogen, AG‐20B‐0042), anti‐METTL3 (Abcam, ab195352), anti‐α‐Tubulin (Proteintech, #11 224), anti‐m^6^A (Beyotime, AF7407), anti‐NLRP3 (Cell Signaling Technology, #15 101), anti‐NEK7 (Abcam, ab133514), anti‐NEK7 (Abcam, ab95873), anti‐ASC (Santa Cruz, sc514414), anti‐NF‐κB‐p65 (Cell Signaling Technology, #8242), anti‐p‐p65 (Cell Signaling Technology, #3033), anti‐TNFAIP3 (Santa Cruz, sc16692), anti‐ubiquitin (Santa Cruz, sc8017), anti‐HA‐Tag (Cell Signaling Technology, #3724), anti‐FLAG‐Tag (Sigma‐Aldrich, #A8592). HRP‐conjugated goat anti‐mouse and goat anti‐rabbit secondary antibodies were obtained from Proteintech (SA00001‐1 and SA00001‐2 respectively). Inhibitors included disulfiram (MedChem Express, HY‐B0240) and COP (TargetMol, CAS: 6020‐18‐4).

### Animal Studies

Mice were kept and bred in the State Key Laboratory of Oral Diseases at Sichuan University under specific pathogen‐free housing conditions. All animal experiments were conducted with the approval by the Subcommittee on Research and Animal Care (SRAC) of Sichuan University (WCHSIRB‐D‐2021‐136; WCHSIRB‐D‐2017‐103). Male C57BL/6J mice were purchased from GemPharmatech LLC. (Chengdu, China). Genetically targeted mice were generated by CRISPR‐Cas9 genome technology. *Mettl3^fl/fl^* C57BL6/J mice were established as previously reported.^[^ ^63^ ^]^ *Tnfaip3^fl/fl^* C57BL6/J were generated by Cyagen Biosciences Lnc. by targeting the exon 3 of mouse *Tnfaip3* gene (GenBank accession number: NM_0 09397.3). *Gli1‐Cre^ER^;Rosa26‐tdTomato* transgenic mice were kindly provided by Dr. Jun Wang from Sichuan University. Genotype identification and lineage tracing were in accordance with previous works. *Mettl3^fl/fl^* mice and *Mettl3^fl/fl^*; *Tnfaip3^fl/fl^* mice were crossed with *Gli1‐Cre^ER^* mice to generate *Gli1‐Cre^ER^; Mettl3^fl/fl^* mice and *Gli1‐Cre^ER^*; *Mettl3^fl/fl^*; *Tnfaip3^fl/fl^* mice, respectively.

For disulfiram experiment, 6‐week‐old male C57BL6/J mice were treated with Cu (II) (0.15 mg kg^−1^) by intraperitoneal injection 6 h before disulfiram administration. Mice were then treated with disulfiram (50 mg kg^−1^) formulated in peanut oil (12.5 mg/ml) or vehicle at indicated times.

For the COP experiment, COP (20 µm) in physiology saline or normal saline was injected into mice buccal gingival of mandibular first molar using a 32G needle after oral ligatures at indicated times.

### Periodontitis Models

Experimental murine periodontitis was induced by oral ligature according to published researches.^[^ ^10^ ^]^ In brief, mice were first anesthetized with xylazine (10 mg kg^−1^) and ketamine (80 mg kg^−1^). Ligatures were placed on the mandibular first molar using 6‐0 silk sutures with knots tying on the buccal side. Sutures were immediately removed in mice with sham treatment. At the endpoints of each experiment, mice heart was perfused with 4% paraformaldehyde (PFA) after anesthesia. Mandibles were carefully dissected and fixed in 4% PFA at 4 °C overnight. After thoroughly washing off with phosphate buffer, samples were subjected for CBCT scanning.

### Micro‐CT Analysis

The fixed mandible samples were scanned by a µCT50 (SCANCO Medical, Bruettisellen, Switzerland) with a spatial resolution of 5 medium solution. The alveolar bone was defined as the region of interest (ROI) and subjected to 3D reconstruction. For the analysis of bone destruction, images of both the lingual reconstruction and the coronal cross‐section were captured by SCANCO Visualizer software (1.1.18.0). Measurements were performed using ImageJ software.

### Histological Staining

Mandible samples were decalcified in 10% ethylenediaminetetraacetic acid (EDTA, pH 7.6) for 4 to 8 weeks after micro‐CT reconstruction. Samples for lineage tracing were immersed in 20% sucrose solution at 4 °C overnight and embedded in OCT compound to prepare frozen sections (10 µm). For paraffin sections (5 µm), samples were dehydrated using degraded ethanol and xylene in accordance with previous work.^[^ ^64^ ^]^

For hematoxylin‐eosin (HE) staining, paraffin sections were conventionally dewaxed to water through xylene and degraded ethanol. Sections were successively stained with hematoxylin and eosin according to the manufacturer's protocol.

For immunohistochemistry (IHC), paraffin sections were dewaxed and rehydrated as described above. Assays was performed using IHC staining kit (Boster Biological Technology, Wuhan, China). In brief, sections were thoroughly immersed in sodium citrate buffer at 95 °C for 10 min and incubated with 3% hydrogen peroxide at room temperature for 10 min. After blocking, sections were incubated with primary and secondary antibodies. Positive signals were detected by DAB or AEC staining kit (Boster Biological Technology).

For immunofluorescence (IF) staining, the deparaffinized sections were incubated with primary antibodies after antigen retrieval, followed by Alexa Fluor 488/550‐conjugated secondary antibodies. Nuclei were detected using DAPI, and images were captured by confocal microscopy (Olympus FV3000).

### Western Blot and Co‐Immunoprecipitation

The protein lysates from mouse gingival tissues and cells were extracted on ice using a protein assay kit (SAB, PE001). Western blotting (WB) was conducted as described previously.^[^ ^65^ ^]^ In brief, equivalent proteins were separated in 10% or 12% sodium dodecyl sulfate‐polyacrylamide (SDS) gels (Bio‐rad) and transferred to polyvinylidene difluoride (PVDF) membranes. Membranes were then blocked in 5% silk milk for 1 h at room temperature. Primary antibodies binding were accomplished at 4 °C overnight. After incubation with secondary HRP‐conjugated antibodies, signals were visualized by the gel imaging system. For co‐immunoprecipitation, cells were lysed in MLB Lysis buffer with protease inhibitor cocktail (Roche Diagnostics, Rotkreuz, Switzerland) for 30 min on ice. Lysates were subsequently centrifuged at 13000 rpm for 15 min at 4 °C. Supernatant was collected and mixed with 10 µg of indicated antibodies at 4 °C overnight. Samples were then incubated with Protein A/G Magnetic Beads (ThermoFisher Scientific) at 4 °C for 2 h. Following washing, the coprecipitated complex was eluted with SDS‐loading buffer at 95 °C for 5 min. Samples were collected for WB analysis.

### Cell Culture

The human gingival fibroblast (HGF) collection procedure was approved by the Ethics Committee of West China Hospital of Stomatology, Sichuan University (WCHSIRB‐D‐2018‐010). HGFs were isolated from gingival tissues of healthy donors under standard protocols. Tissues collected from teeth‐lengthening surgeries were immediately stored in phosphate‐buffered saline (PBS) on ice and transferred to the inoculating room. Gingival tissues were washed 8–10 times in PBS. Following washing, tissues were incubated with a Dispase II solution (4 mg/ml) for 1 h at 37 °C to separate the stromal layer. Then next incubated the tissues with 2 mg/ml collagenase I solution for 1 h at 37 °C with gentle shaking and filtered the mixture through a 40 µm cell mesh filter (Falcon, BD Bioscience, NJ, USA). HGFs were resuspended in fresh DMEM medium (Hyclone Laboratories, USA) containing 20% v/v fetal bovine serum (Gibco, USA), 100U/ml penicillin and 100 µg ml^−1^ streptomycin (Gibco, USA). HGFs of passage 3 (P3) to passage 7 (P7) were used in the following experiments. The iBMDM cell line was a gift from Professor Feng Shao. HEK‐293T cells and iBMDMs were cultured in DMEM medium with 10% FBS v/v. For compounds selection, MOLM‐13 cells obtained from ATCC were cultured in RPMI‐1640 medium with 10% FBS v/v.

### siRNA and Plasmids Transfection

Gene knockdown in HGFs was induced by siRNA transfection. The siRNAs for METTL3, TNFAIP3, and scramble control were synthesized by Sangon Biotech (Shanghai, China). The sequences were as follows: human METTL3‐siRNA sense: CUGCAAGUAUGUUCACUAUGA; human TNFAIP3‐siRNA‐1 sense: CAAAGCACUUAUUGACAGA; human TNFAIP3‐siRNA‐2 sense: AGAGACAUGCCUCGAACU; human TNFAIP3‐siRNA‐3 sense: GCACCUAAGCCAACGAGU; mouse METTL3‐siRNA sense: GCUACCGUAUGGGACAUUAUU. HGFs and iBMDMs were transfected using Lipofectamine RNAimax (Invitrogen, USA) according to the manufacturer's instructions. Quantitative Real‐time PCR (qRT‐PCR) and western blot assay were established to test knockdown efficiency.

For TNFAIP3 knockout (KO), HGFs or iBMDMs were transfected with CRISPR/Cas9 KO plasmids (sc‐400447‐KO‐2) obtained from Santa Cruz (Dallas, Texas). The Tnfaip3‐Flag, Nek7‐HA, and Ub‐His plasmids were cloned into pCDNA 3.1 vectors by GeneralBio (Chuzhou, China). The delivery of plasmids was carried out with Lipofectamin 3000 transfection reagent following the manufacturer's protocols.

### RNA Sequencing and Analysis

Total RNAs from pro‐inflammatory treated HGFs with or without METTL3 knockdown were extracted by Trizol reagent and quantified by the NanoPhotometer spectrophotometer (IMPLEN, USA). RNA integrity was estimated by Agilent Bioanalyzer 2100 (Agilent Technologies, USA). Library for transcriptome sequencing was prepared using NEBNext Ultra RNA Library Prep Kit (NEB, USA) as previously described.^[^ ^66^ ^]^ Poly‐T oligo‐attached magnetic beads were used to purify mRNA. Briefly, we used FastQC (v0.11.5) and FASTX toolkit (0.0.13) for the quality control of raw data, which were subsequently mapped to Homo sapiens reference genomes using HISAT2 (v.2.0.5). FeatureCounts (v1.5.0) and DESeq2 R package (1.16.1) were performed to identify gene differential expression. The threshold set differentially regulated genes was a fold change ≥ 2.0 and a P value ≤ 0.05. Afterward, GO analysis and KEGG analysis were implemented to determine the intrinsic roles of these differentially expressed transcripts. For GSEA, gene sets for innate immunity and inflammasome pathway were obtained from GSEA online database and a published study, respectively. GSEA software was used to align our gene lists of interest and the given gene sets. Further KEGG and GO analysis were performed using Metascape (metascape.org).^[^ ^67^ ^]^

### Analysis of MeRIP Data

MeRIP‐seq data are from the published MeRIP‐sequencing dataset GSE162469, GSE152042 and GSE145315. Sequence reading and mapping was performed, visualization of peaks, metagene analysis depicting the distribution of m6A modifications, motif searching, and ontology analysis in accordance with previously established methodologies.^[^ ^68^ ^]^

### Quantitative Real‐Time PCR

Total RNAs were extracted using TRIzol (Invitrogen) following the manufacturer's instructions. Complementary DNA (cDNA) was prepared by TaqMan Reverse Transcription Reagents (Takara) as previously described.^[^ ^39^, ^66^ ^]^ Genes were amplified and analyzed by Real‐time PCR using Bio‐Rad SYBR Green Premix Ex Taq (Takara) in a Roche LightCycler 96 system.

### ELISA of IL‐1β

The quantity of IL‐1β in mouse serum or cell culture supernatants was measured by mouse IL‐1β ELISA kit and human IL‐1β ELISA kit (NeoBioscience, China), respectively. ELISA assays were implemented following the manufacturer's instructions.

### LDH Release Assay

LDH release assay was performed using a non‐radioactive cytotoxicity assay kit (Promega). Cells were cultured on a 96‐well plate and prepared with designated treatment. Cell maximum LDH release was induced by adding the lysis solution and cytotoxicity was measured according to the manufacturer's instructions.

### PI Uptake Staining

HGFs were transfected with siMETTL3 or siCTRL prior to pro‐inflammatory induction. PI staining was performed following the manufacturer's protocols (Solarbio, China). In brief, cells were dyed with PI and Hoechst 33 342 solution for 30 min at 4 °C. Images were captured by microscopy.

### LC‐MS/MS

The NEK7 protein and its interacting proteins were coimmunoprecipitated and subjected to LC‐MS/MS assay. The following procedures were carried out by PTM Biolabs Co., Ltd. (Hangzhou, China). In brief, the proteins were denatured and alkylated in SDS solution, which were determined using Bradford analysis prior to SDS‐PAGE detection. Amino peptides were labeled with isobaric iTRAQ tags for further separation by strong citation exchange choematography. iTRAQ labeling and the masses were examined by an LC‐MS/MS Q Exactive Hybrid Quadrupole‐Orbitrap Mass Spectrometer (Thermo Finnigan, San Jose, CA). iTRAQ Result Multiple File Distiller was used to process raw data for quantification (FDR ≤ 0.01 for data filtration).

### Dot Blot

HGFs were primed with *E.coli*. derived LPS for 24 h and activated by 4 mM ATP for 30 min. The total RNA of HGFs and MOLM‐13 cells was extracted as mentioned above. An m^6^A dot blot assay was subsequently performed to measure the m^6^A amount according to a published protocol. In brief, mRNAs were purified and diluted to obtain 250 and 25 ng µl^−1^ samples. After denaturation at 95 °C for 3 min, mRNAs were chilled on ice immediately. A 2 µl drop of sample was loaded onto the Hybond‐N+ membrane and crosslinked under ultraviolet radiation. Membranes were blocked in 5% silk milk for 1 h at room temperature and incubated with the m^6^A antibody (Beyotime, China) at 4 °C overnight. For the next day, membranes were incubated with the secondary antibody for 1 h. Signals were captured using the ChemiDoc imaging system (Biorad, USA). Finally, the membranes were soaked in 0.02% methylene blue in 0.3 M sodium acetate (pH 5.2) to show load control.

### RNA Stability

Cells were seeded on 6‐well plates with 10^6^ per well and transfected with siCTRL or siMETTL3. To assess *TNFAIP3* stability, 5 µg ml^−1^ Actinomycin D was added to cells. Samples were collected at 0, 4, 8, and 12 h after termination. RNA was extracted and examined using real‐time qPCR.

### In‐Vitro Ubiquitination

In‐vitro ubiquitination assay was performed using a Ubiquitinylation Kit (Enzo, BML‐UW9920‐0001) following the procedure instructions. After the reaction, immunoprecipitation of NEK7 with an anti‐NEK7 antibody (Abcam, ab95873) was performed. Samples were mixed with SDS‐loading buffer at 95 °C for 5 min for WB analysis.

### MTT Assay

Cells were treated with various concentrations of compounds for 3 days. 20 µl 5 mg/ml MTT (Sigma) solution (5 mg/ml MTT in 0.9% NaCl) was added to each well to stop treatment. After 1 to 4 h incubation in CO_2_ incubators, remove the media with needles and add 150 µl DMSO (Sigma, Cat. No. D2650) to each well. To dissolve crystals, plates were gently shaken at RT for 15–25 min. Transfer plates to plate reader and measure absorbance at the wavelength of 570 nm.

### Molecular Docking‐Based Virtual Screening

The 3‐D structure of METTL3 was taken from the crystal structure of METTL3 in a complex with S‐Adenosyl‐L‐methion (SAM) (PDB ID 5K7U). The receptor structure was prepared by the Discovery Studio (DS) 3.1 (Accelrys Inc., San Diego, CA, USA) software package with the standard preparation procedure (protein preparation protocol). This procedure encompassed the removal of water molecules and METTL14, the addition of hydrogen atoms to the protein, and the assignment of force field parameters, with the adoption of the CHARMm force field. In this research, the molecular docking method GOLD was used, and the “pre‐defined generic algorithm (GA)” setting of “7‐8 times speed up” and “automatic” was employed and other parameters were set to default. Compound libraries used for virtual screening in this investigation include the Vitas‐M chemical library (≈1.5 m compounds, Vitas‐M Laboratory, Ltd) and an in‐house chemical library (35000 compounds). All the compounds used for docking were pre‐screened by PAINS and Lipinski Rules of Five.^[^ ^69^ ^]^ Selected compounds were purchased in milligram quantities from Vitas‐M Laboratory and TargetMol (Shanghai, China). The purity of the compounds was ≥ 95%, as declared by chemical vendors.

### Protein Expression and Purification

Proteins were ectopically over‐expressed and purified as described previously.^[^ ^70^ ^]^ The expression vector of the full‐length METTL3 was constructed by inserting the BamHI/HindIII sites of pET28a(+)(Genewiz) and those encoding human METTL3(1‐591) with a hexahistine‐tag at the N‐terminal. Specified constructs of human METTL3 and METTL14 were subcloned into pETDuet vector and transformed into Rosetta (DE3) pLysS cells (Novagen).^[^ ^70^ ^]^ The protein expression vector was transfected into E. coli BL21 (DE3) competent cells. Induction of protein expression was achieved by supplementing the culture with 0.1 mm IPTG at the logarithmic growth phase, followed by overnight incubation at 18 °C. The cultures were harvested and broken in lysis buffer (25 mm Tris‐HCl, pH 8.0, 150 mm NaCl, and 1 mm phenylmethanesulfonyl‐fluoride (PMSF)). The suspension was sonicated in an ice bath, following centrifugation at 16,000 rpm at 4 °C for 45 min. The supernatant was loaded onto an Ni‐NTA affinity column (QIAGEN) and the beads were washed two times with a tenfoldvolume of lysis buffer, followed by three times washing with 10 mM imidazole (5 column‐volumes each). Protein elution was accomplished using 250 mm imidazole in lysis buffer. The protein was further purified to homogeneity by ion‐exchange chromatography and size‐exclusion column (Supedex 200 increase, GE Healthcare) equilibrated with 20 mm Tris‐HCl buffer (pH 8.0) and 100 mm NaCl. The purified protein was frozen in aliquots and stored in liquid nitrogen until use. The purities of these proteins were confirmed by SDS‐PAGE.

### Differential Scanning Fluorimetry (DSF) Assays

DSF experiments were executed in a RT‐PCR detection system (BIO‐RAD CFX96) according to the protocol.^[^ ^71^ ^]^ Signal of SYPRO orange (Sigma, S5692) was detected utilizing FRET filters with excitation at a wavelength of 492 nm and ROX filters with emission at a wavelength of 610 nm. Each reaction solution contained 2 µm METTL3 proteins, 5 × SYPRO orange, and tested compounds in 10 µL buffer (20 mm Hepes, PH 7.4, and 100 mm NaCl) and heated from 25 to 95 °C. Fluorescence intensities were recorded at intervals of 1 °C min^−1^. The inflection point of the transition curve (Tm) was calculated by fitting the Boltzmann equation to the sigmoidal curve in GraphPad Prism 5.0.

### Inhibition of m^6^A Methylation in RNAs

Based on the known protocol,^[^ ^72^ ^]^ reactions were consistently conducted on the same scale described for the inhibition of labeled oligo DNA/RNA chimera fragments methylation, except for using oligo DNA/RNA (5′‐FAM – d(CAT) r(GGACA) d(TATGT) – 3′ BHQ‐1) at 1 µm and METTL3/14 protein at 2.5 µm. Labeled oligo RNA or DNA/RNA chimera fragments (1 µm) were mixed with METTL3/METTL14 in the methylation buffer (25 mm Tris‐HCl, 0.01% Tween‐20, 1 mm DTT, 50 µm ZnCl2, 0.1 U µL^−1^ RNase Inhibitor (Takara, 2313A), and 10 µm SAM, pH 7.5) containing various concentrations (threefold dilution) of Coptisine chloride (COP). The above solution was reacted at 25 °C for 60 min and at 95 °C for 3 min. Finally, one‐fifth of the sample volume underwent the MazF reaction for subsequent FRET measurements.

### Surface Plasmon Resonance (SPR) Assays

SPR technology‐based binding assays were carried out using a Biacore X100 instrument (GE Healthcare). The immoblilized METTL3 proteins on a CM5 sensor chip using a standard amide coupling procedure within 10 mm sodium acetate buffer (pH 4.5). Protein solution of 100 µg ml^−1^ were added to react with the activated surface. The chip was equilibrated with 1.05x PBS buffer at room temperature for 4 h. Compounds were serially diluted and injected for 60 s (contact phase) at a flow rate of 30 µL min^−1^, followed by 120 s of buffer flow (dissociation phase). The KD values were evaluated by BIA evaluation software (GE Healthcare).

### Microscale Thermophoresis (MST) Assays

MST assays were performed by HJ Century Biopharmaceatical Inc (Wuhan, China). Briefly, METTL3 was labeled with NT647 dye (NanoTemper Technologies) and applied at a final concentration of 340.43 nm in 20 mm HEPES, PH 8.0, and 150 mm NaCl. Twofold diluted Coptisine chloride (COP) in assay buffer was mixed with METTL3 solutions to generate a final compound concentration ranging from 2 mm to 0.83 nm. After 10‐min incubation at room temperature, samples were filled into standard treated capillaries (NanoTemper Technologies), and MST measurements were performed on a Monolith NT.115 (NanoTemper Technologies) using 100% LED and 20% or 40% IR‐laser power. Laser on and off times were respectively set at 30 and 5 s.

### Statistics

Data were expressed as the mean ± standard deviation (SD). Comparisons between groups were evaluated by unpaired two‐tailed Student's *t*‐test or one‐way ANOVA followed by Turkey's or Dunnet's post‐hoc test. A *P*‐value of less than 0.05 was considered to have a statistical difference. GraphPad Prism was used for data statistical analysis.

### Study Approval

All animal experiments were conducted with the approval by the Subcommittee on Research and Animal Care (SRAC) of Sichuan University (WCHSIRB‐D‐2021‐136; WCHSIRB‐D‐2017‐103). The human gingival fibroblast (HGF) collection procedure was approved by the Ethics Committee of West China Hospital of Stomatology, Sichuan University (WCHSIRB‐D‐2018‐010).

## Conflict of Interest

The authors declare no conflict of interest.

## Author Contributions

X.Y.Z. and X.Y.Y. contributed equally to this work and are co‐first authors. Conceptualization: X.Y.Z., X.Y.Y., S.Y.Y., Q.Y., B.S. Methodology: X.Y.Z., X.Y.Y., S.Z.H., G.F.L, K.X.L., Q.W., W.M.L., X.Y.Q. Investigation: X.Y.Z., X.Y.Y., S.Z.H., G.F.L., K.X.L. Visualization: X.Y.Z., X.Y.Y. Funding acquisition: Q.Y., X/Y.Z. Project administration: Q.Y., S.Y.Y., B.S., X.Y.Z. Supervision: Q.Y., B.S., S.Y.Y, D.S. Writing – original draft: X.Y.Z, X.Y.Y, S.Z.H., X.Y.Q. Writing – review & editing: X.Y.Z., X.Y.Y., S.Z.H., G.F.L., K.X.L., Q.W., W.M.L., H.W.L., X.Y.Q., D.S., S.Y.Y., B.S., Q.Y. The order of the first authors is based on the contribution to data collation and manuscript writing.

## Supporting information

Supporting Information

## Data Availability

The data that support the findings of this study are available from the corresponding author upon resonable request.
