# Supplementary material for: Inhibition of METTL3 Alleviates NLRP3 Inflammasome Activation via Increasing Ubiquitination of NEK7
Source: Adv Sci (Weinh). 2024 May 2;11(26):2308786. doi: 10.1002/advs.202308786 (PMC11234428; doi:10.1002/advs.202308786)
Supplement: Supplementary file 1 — Supporting Information [file ADVS-11-2308786-s001.pdf]

ADVANCED SCIENCE
Open Access

Supporting Information

for *Adv. Sci.*, DOI 10.1002/advs.202308786

Inhibition of METTL3 Alleviates NLRP3 Inflammasome Activation via Increasing Ubiquitination of NEK7

Xinyi Zhou, Xiaoyu Yang, Shenzhen Huang, Guifeng Lin, Kexin Lei, Qian Wang, Weimin Lin, Hanwen Li, Xingying Qi, Dutmanee Seriwatanachai, Shengyong Yang, Bin Shao and Quan Yuan**

Inhibition of METTL3 alleviates NLRP3 inflammasome activation via increasing ubiquitination of NEK7

Xinyi Zhou*, Xiaoyu Yang*, Shenzhen Huang, Guifeng Lin, Kexin Lei, Qian Wang, Weimin Lin, Hanwen Li, Xingying Qi, Dutmanee Seriwatanachai, Shengyong Yang, Bin Shao†, Quan Yuan†

Supporting Information

Figure S1. Identification of genotypes and knockout efficiency. 2

Figure S2. The administration of STM2457 alleviates periodontal inflammation. 3

Figure S3. RNA-sequencing analysis of HGFs. 4

Figure S4. Western blot analysis of periodontal gingival tissues. 4

Figure S5. METTL3 installs m^{6}A modification on *Tnfaip3* mRNA. 5

Figure S6. The METTL3-TNFAIP3-NEK7 axis exists within macrophages. 6

Figure S7. Coptisine chloride (COP) selectively inhibit methyltransferase function of METTL3. 7

Figure S8. Predicted binding model of COP and METTL3. 8

Table S1. ΔT_m values of 30 hit compounds measured in the DSF assays 9

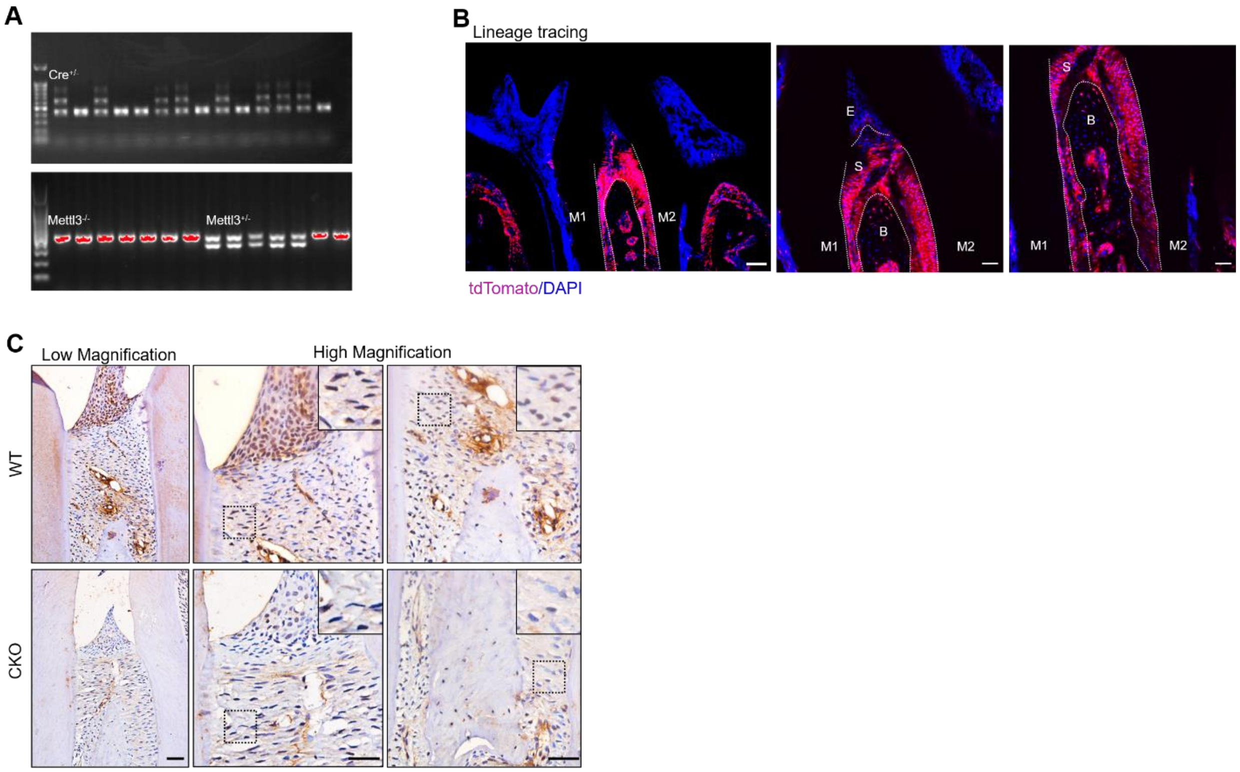

Figure S1. Identification of genotypes and knockout efficiency.

A. Genotyping PCR and southern blot analysis of *Cre* and *Mettl3*. For *Cre* identification, Mut=590 bp and WT=410 bp; For *Mettl3* identification, Mut=391 bp and WT= 300 bp. B. Representative images of Gli1+ cells and their descendants in periodontal tissues. The middle and the right panel showed enlarged images of periodontal stroma layer and periodontal ligaments, respectively. Scale bar, 20 μm. C. Immunohistochemical staining of METTL3 in *Gli1-Cre*^{ERT2}*;Mettl3*$^{fl/fl}$ mice. Scale, 20 μm.

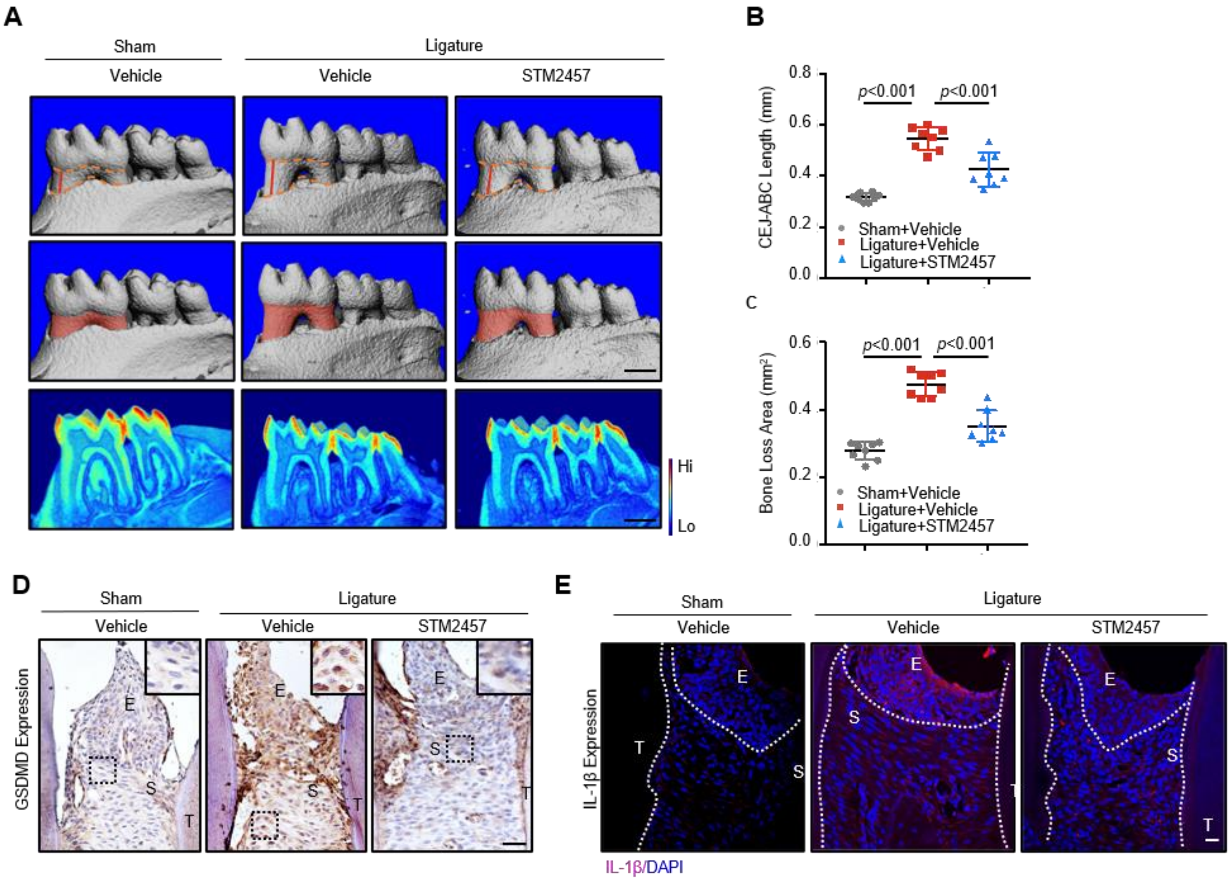

Figure S2. The administration of STM2457 alleviates periodontal inflammation.

A. Micro-CT analysis and 3D reconstructed images observed from lingual side. Mice (6-week-age, male) were oral-ligated and treated with vehicle or STM2547(50mg/kg/d). B. Quantification of the distance between CEJ-ABC. C. bone loss area in 3 groups. n= 8 biological replicates, one-way ANOVA. D. Representative IHC staining and quantification of GSDMD expression in periodontal tissues in 3 groups. Scale bars, 50 μm. E. Representative IF staining of IL-1β expression in periodontal tissues from 3 groups. Purple signals are for IL-1β positive staining and blue signals indicate DAPI. Scale bars, 20 μm. Values are shown as mean ± SD.

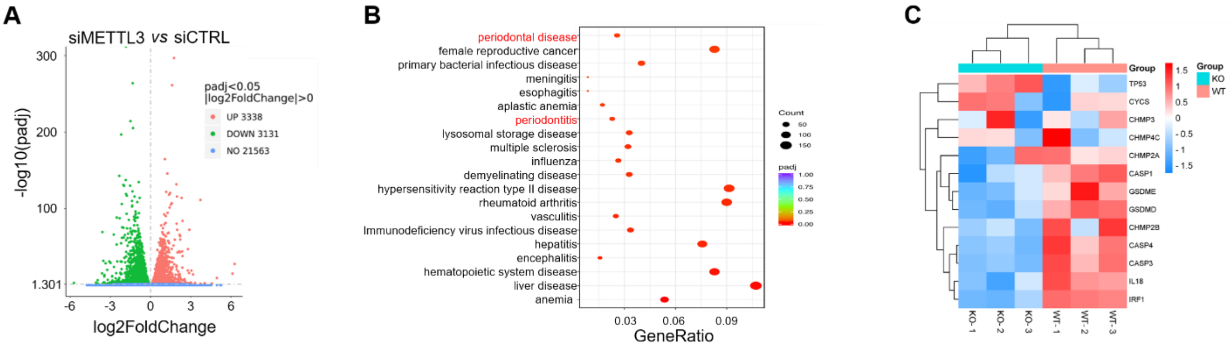

Figure S3. RNA-sequencing analysis of HGFs.

A. Volcano plot of RNA-seq data from HGFs transfected with siMETTL3 or siCTRL. Each point represents an individual gene with FDR＜0.05. All differentially expressed genes are highlighted in red (up-regulated) or green (down-regulated). B. GO analysis for down-regulated differentially expressed genes. C. Heatmap of representative genes involved in pyroptosis pathway.

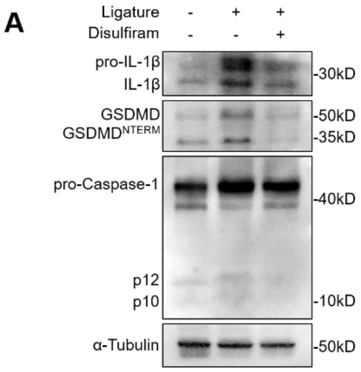

Figure S4. Western blot analysis of periodontal gingival tissues.

A. Western blot analysis of GSDMD, IL-1β and caspase-1 protein expression in mouse periodontal gingival tissues.

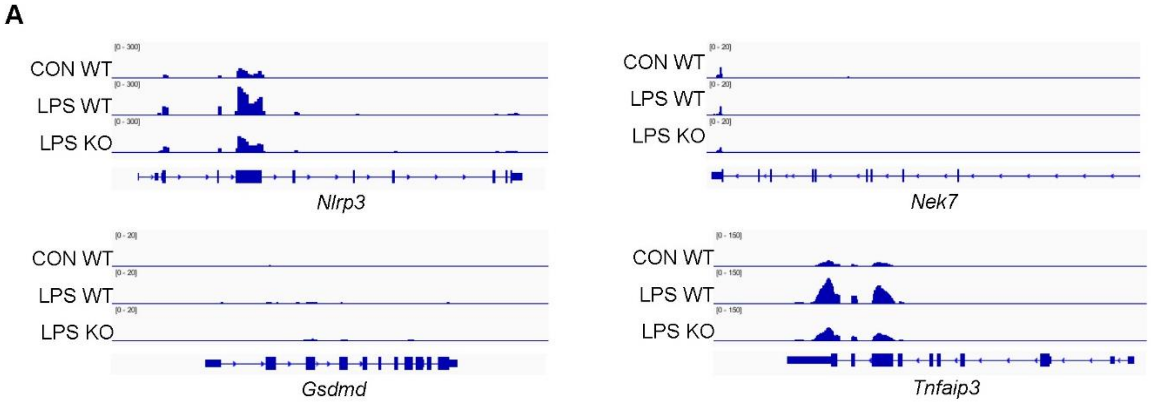

Figure S5. METTL3 installs m^{6}A modification on *Tnfaip3* mRNA.

A. m^{6}A MeRIP-Seq on Raw 264.7 cells with control treatment or LPS stimulation. WT for wild type Raw 264.7 cells and KO for *Mettl3*-KO Raw 264.7 cells.

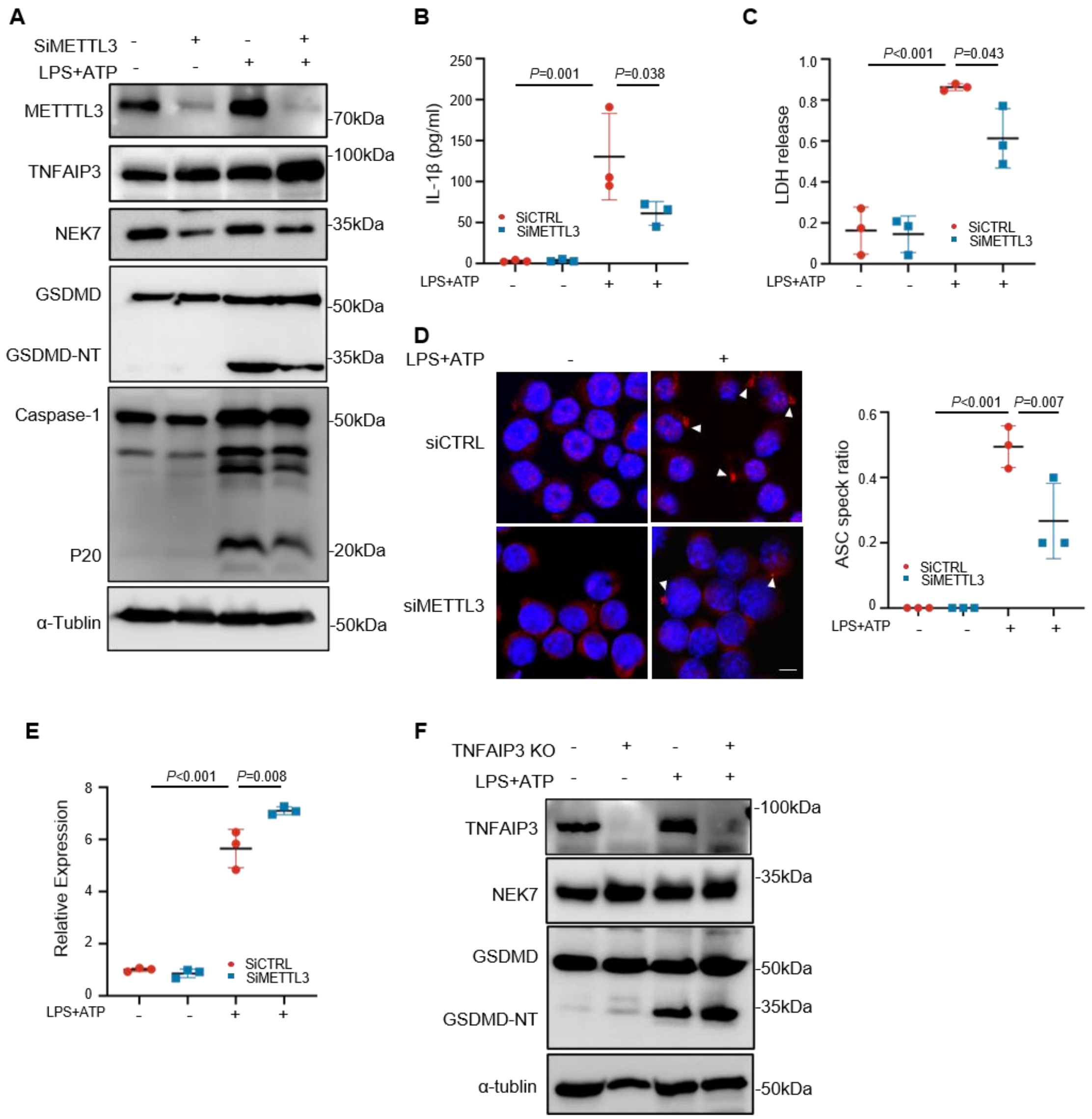

Figure S6. The METTL3-TNFAIP3-NEK7 axis exists within macrophages.

A. Western blot analysis of TNFAIP3, NEK7, GSDMD and Caspase-1 in iBMDMs. B-C. Release of IL-1β and LDH in cultured supernatants of iBMDMs (n=3, unpaired two-tail Student's *t*-test). D. IF staining and quantification of ASC speck in iBMDMs (n=3, unpaired two-tail Student's *t*-test). Scale bars, 20 μm. E. Quantitative real-time PCR analysis of *TNFAIP3* (n=3, unpaired two-tail Student's *t*-test). F. Western blot analysis of NEK7 and GSDMD in *TNFAIP3* knockout (KO) iBMDMs.

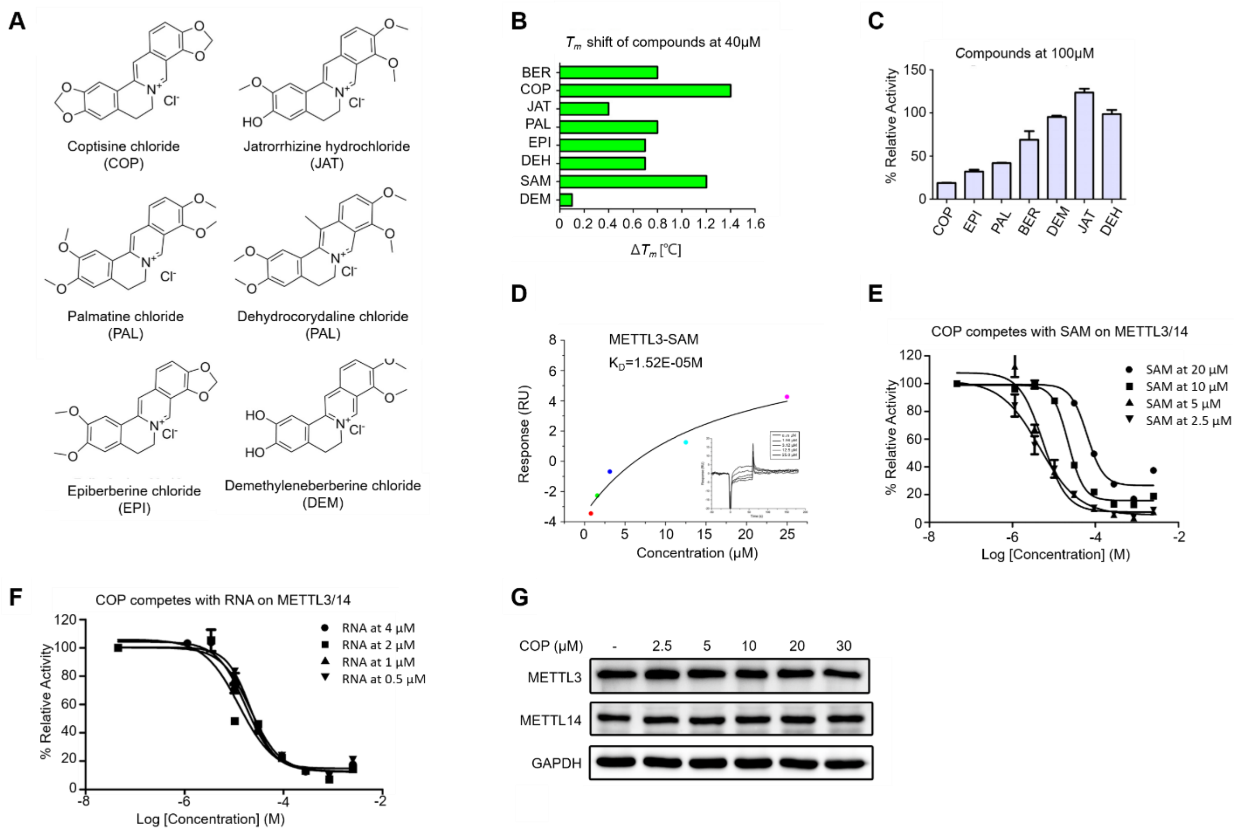

Figure S7. Coptisine chloride (COP) selectively inhibit methyltransferase function of METTL3.

A. Chemical structures of the six analogues of BER. B. T_m shifts of compounds at 40 µM in the DSF assays. C. METTL3/14 methyltransferase inhibition activities of compounds at 100 µM in the FRET-based MazF assays. D. Representative METTL3 binding curves and fit steady-state evaluation for SAM using SPR. E,F. Kinetic experiment results. G. Western blot analysis of METTL3 and METTL14 protein expression in MOLM-13 cells received COP treatment.

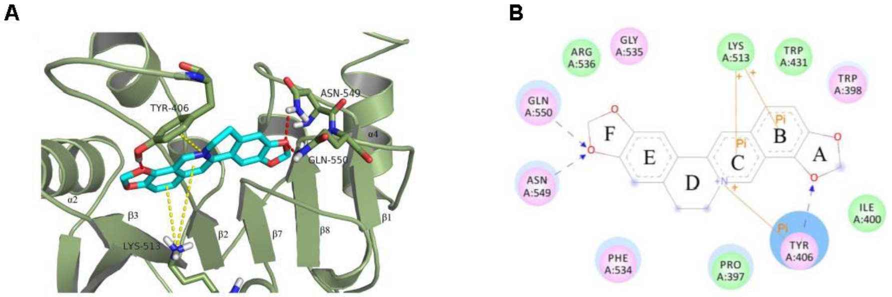

Figure S8. Predicted binding model of COP and METTL3.

A. Three-dimensional docking model of COP binds in the active pocket of METTL3. B. Two-dimensional diagram of COP and the key residues involved in binding with METTL3.

Table S1. ΔT_m values of 30 hit compounds measured in the DSF assays

NO	IDNUMBER	Structure	ΔT_m (℃)
1	STK499370	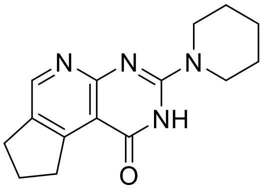	-0.58
2	STK211475	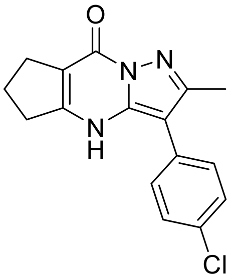	0.38
3	STK169704	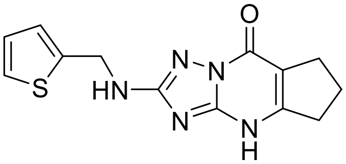	0.26
4	STK506346	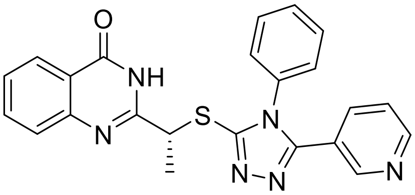	0.37
5	STK901648	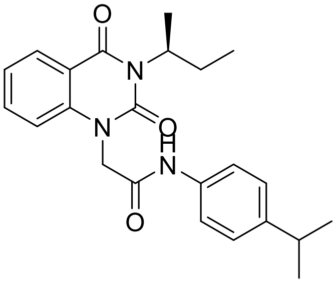	0.06
6	STL191227	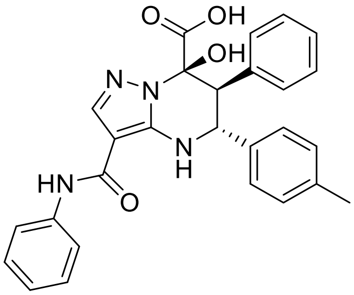	0.28

7	STL175076	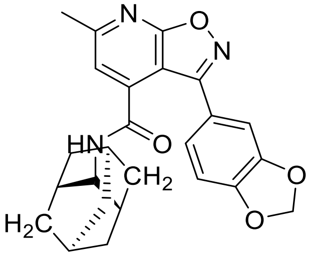	-0.13
8	STL008354	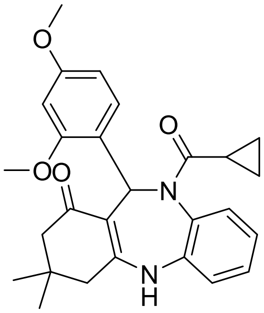	0.35
9	STK057060	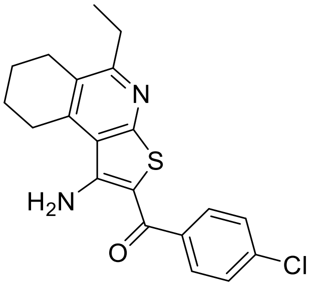	0.17
10	STK352385	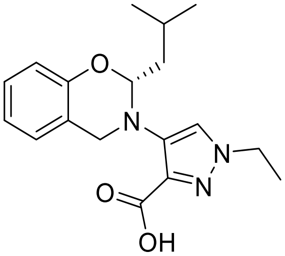	0.23
11	STK007445	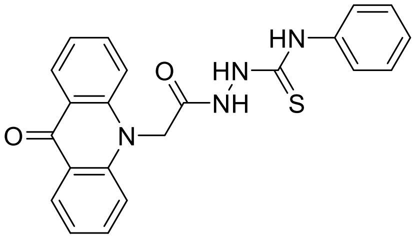	0.53
12	STK397150	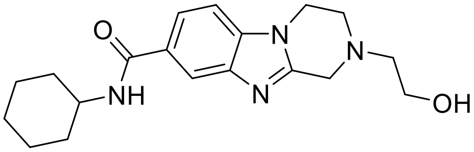	0.46

13	STL099392	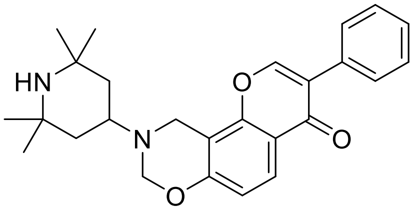	0.37
14	STK289134	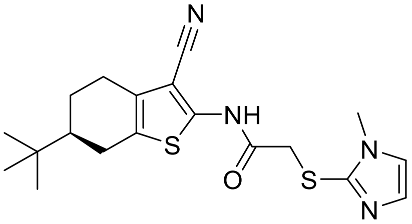	0.16
15	STK166791	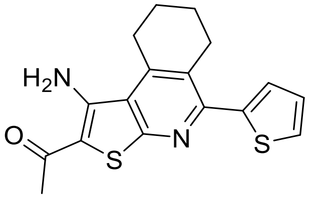	-1.78
16	STK256723	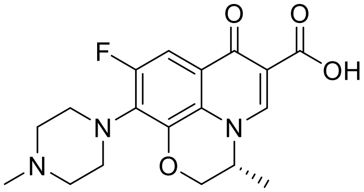	0.20
17	STK525355	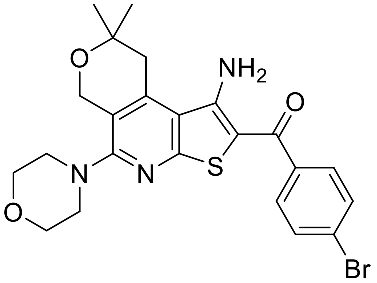	0.36
18	STK539456	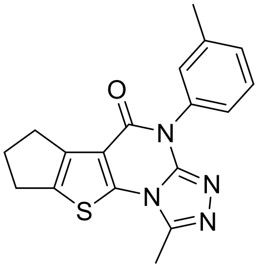	0.15
19	STL044251	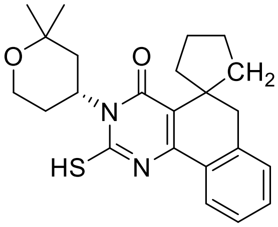	0.24

20	STL242889	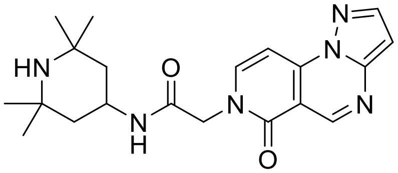	0.52
21	STL312325	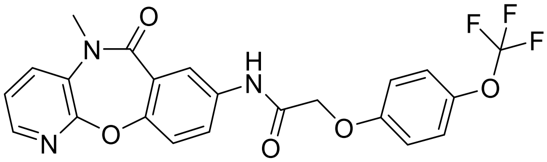	0.17
22	STL348275	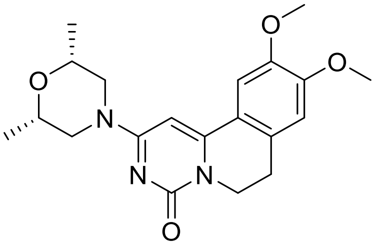	-0.74
23	STL059197	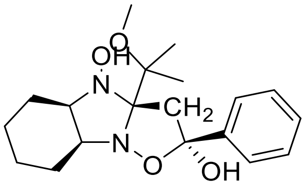	0.37
24	STK801617	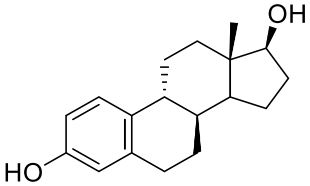	0.06
25	STK526460	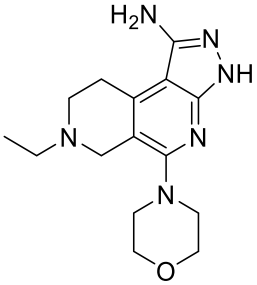	-0.17
26	STK057618	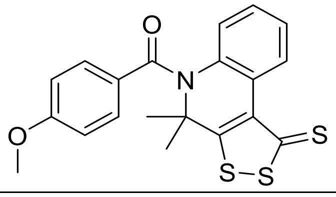	0.53
27	STK261312	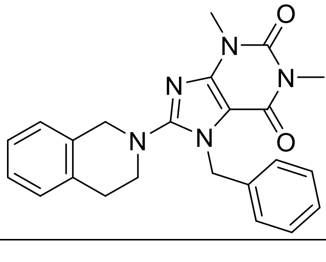	0.35

28	STK081465	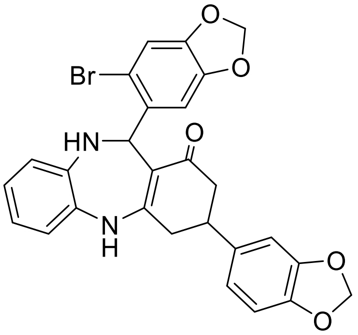	0.06
29	STK020495	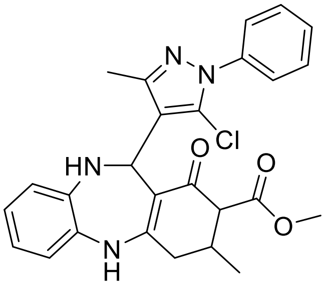	-0.03
30	STK870320 (Berberine hydrochloride)	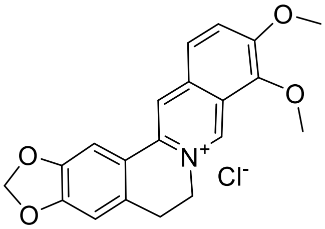	0.61
31	S-Adenosyl-L-methionine (SAM)	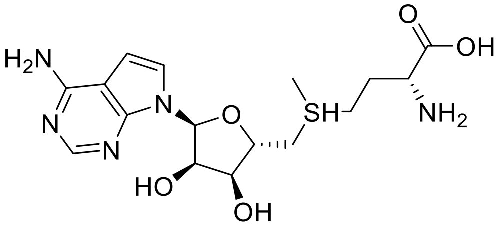	0.98
